# OmicsTransformer: self-supervised masked consistency and uncertainty-aware fusion for robust multi-omics prediction

**DOI:** 10.1093/bioinformatics/btag468

**Published:** 2026-06-29

**Authors:** Junxuan Feng, Bingshen Shan, Jie Deng, Zixin Jiang, Siqin Peng, Sijun Peng, Jian Yang, Gang Wang, Xiaogang Peng, Xiaozheng Li

**Affiliations:** College of Computer Science and Software Engineering, Shenzhen University, Shenzhen, 518060, China; National Engineering Laboratory for Big Data System Computing Technology, Shenzhen University, Shenzhen, 518060, China; College of Computer Science and Software Engineering, Shenzhen University, Shenzhen, 518060, China; National Engineering Laboratory for Big Data System Computing Technology, Shenzhen University, Shenzhen, 518060, China; College of Computer Science and Software Engineering, Shenzhen University, Shenzhen, 518060, China; National Engineering Laboratory for Big Data System Computing Technology, Shenzhen University, Shenzhen, 518060, China; College of Mechatronics and Control Engineering, Shenzhen University, Shenzhen, 518060, China; School of Computer Science, Wuhan University, Wuhan, 430072, China; Zhejiang University–University of Edinburgh Institute, Zhejiang University, Hangzhou, 314400, China; Beijing Key Laboratory of Mental Disorders, National Clinical Research Center for Mental Disorders and National Center for Mental Disorders, Beijing Anding Hospital, Capital Medical University, Beijing, 100088, China; Advanced Innovation Center for Human Brain Protection, Capital Medical University, Beijing, 100088, China; Beijing Key Laboratory of Mental Disorders, National Clinical Research Center for Mental Disorders and National Center for Mental Disorders, Beijing Anding Hospital, Capital Medical University, Beijing, 100088, China; Advanced Innovation Center for Human Brain Protection, Capital Medical University, Beijing, 100088, China; National Engineering Laboratory for Big Data System Computing Technology, Shenzhen University, Shenzhen, 518060, China; College of Life Sciences and Oceanography, Shenzhen University, Shenzhen, 518060, China

## Abstract

**Motivation:**

Multi-omics integration can improve cancer diagnosis and prognosis, but current models are limited by extreme dimensionality, redundant raw-feature similarities, missing assays, and incomplete pathway priors. We ask whether biologically meaningful patient manifolds can be learned directly from high-dimensional multi-omics data without heuristic graph construction or fixed knowledge-base constraints.

**Results:**

We present OmicsTransformer, an end-to-end framework that projects each omics modality into latent patches, enforces masked semantic consistency through an Exponential Cosine Consistency Loss, models global patch dependencies with a Transformer encoder, and fuses modalities by sample-specific uncertainty. Across eight diagnostic and prognostic cohorts, OmicsTransformer achieved strong performance, including 89.4% accuracy for TCGA-BRCA subtyping and 90.6% area under the receiver operating characteristic curve (AUC) for TCGA-LGG grading. It improved recurrence prediction over the pathway-restricted DeepKEGG baseline by approximately 21.5 percentage points in accuracy (ACC) on TCGA-LIHC and 11.1 percentage points in ACC on TCGA-BLCA. Variance-weighted attribution with ensemble stability selection recovered reproducible cross-modal biomarker cores and non-canonical progression drivers.

**Availability and implementation:**

Source code and datasets are freely available at https://github.com/FFJXX/OmicTransformer and https://doi.org/10.6084/m9.figshare.31523905. OmicsTransformer is implemented in PyTorch.

## 1 Introduction

High-throughput sequencing now enables routine molecular profiling of tumours across multiple layers—somatic variation, gene expression, and epigenetic regulation—creating the opportunity for precision oncology models that operate on the molecular phenotype rather than histology alone. Because each omic layer captures a distinct aspect of tumour biology, joint modelling can improve both diagnostic stratification (subtyping and grading) and prognostic risk estimation such as recurrence prediction, which remains a major driver of cancer mortality (Melo *et al.* 2013, Ren *et al.* 2016, Chakraborty *et al.* 2018, Wang *et al.* 2021b, Wei *et al.* 2023, Cao *et al.* 2024, Yang *et al.* 2025).

Multi-omics learning is, however, intrinsically ill-posed. Feature spaces are orders of magnitude larger than cohort sizes, measurements are noisy and partially missing, and many informative signals are encoded in correlated modules rather than in individual features. In such regimes, classical distances and similarities concentrate and become poorly discriminative, destabilizing topology construction and clustering (Aggarwal *et al.* 2001). The problem is further compounded in clinical practice, where patients frequently lack one or more assays due to cost or specimen constraints.

Early integration methods sought to project modalities into a shared latent space using multiview statistics such as kernel canonical correlation analysis (CCA) and its deep variants (Lai and Fyfe 2000, Andrew *et al.* 2013, El-Manzalawy 2018, Qi *et al.* 2021, Jiang *et al.* 2023). Although effective at capturing coarse cross-modal correlations, these approaches assume relatively simple linear relationships and do not model the higher-order dependencies required for complex phenotypes such as recurrence.

Deep learning has consequently become the dominant integration paradigm. A widely adopted data-driven strategy—exemplified by MOGONET, MOGLAM, and CLCLSA (Wang *et al.* 2021a, Ouyang *et al.* 2023, Zhao *et al.* 2024)—is to construct patient similarity graphs and apply graph convolutional networks for joint representation learning and classification. More recent methods such as MMdynamic (Han *et al.* 2022) have improved fusion reliability through confidence estimation at the decision level. Closely related multiview subspace clustering methods, which similarly construct affinity graphs from multi-view data under low-rank constraints (Lan *et al.* 2024b), highlight that graph topology quality critically determines downstream clustering fidelity. Nevertheless, graph-based pipelines remain vulnerable to the redundancy of raw omics profiles: when pairwise similarities are uniformly high—as we observe across all cohorts studied here (mean cosine similarity 96%–99%; Table 1, available as supplementary data at *Bioinformatics* online)—heuristic graphs become dense and noisy, introducing spurious edges that obscure genuine biological heterogeneity. This limitation reflects the well-characterized behaviour of similarity metrics in high-dimensional spaces (Aggarwal *et al.* 2001).

**Table 1:** Summary of multi-omics datasets and tasks used to evaluate OmicsTransformer.^a^

Category	Dataset	Sample size	Features (multi-omics dimensions)	Task
Diagnostic Cohorts (Disease Subtyping/Grading)	TCGA-BRCA	875	1000 mRNA 1000 DNA methylation 503 miRNA	Five-class subtype classification
	TCGA-LGG	510	2000 mRNA 2000 DNA methylation 548 miRNA	Binary grade classification
	ROSMAP	351	200 mRNA 200 DNA methylation 200 miRNA	Binary AD versus Control
Prognostic Cohorts (Tumour Recurrence Prediction)	TCGA-BLCA	408	1000 mRNA 1000 SNV 100 miRNA	Prognosis of Bladder Urothelial Carcinoma
	TCGA-LIHC	354	1000 mRNA 1000 SNV 200 miRNA	Prognosis of Liver Hepatocellular Carcinoma
	TCGA-PRAD	250	1500 mRNA 2000 SNV 100 miRNA	Prognosis of Prostate Adenocarcinoma
	TARGET-WT	102	2000 mRNA 200 miRNA	Prognosis of Wilms Tumour
	TCGA-BRCA (Recurrence)	211	1000 mRNA 1000 SNV 100 miRNA	Prognosis of recurrence status (subset of BRCA)

a Three diagnostic and five prognostic cohorts are included in the study, detailing sample sizes, multi-omics feature dimensions, and specific classification tasks.

An alternative direction injects biological priors by constraining model connectivity to curated pathway databases. DeepKEGG, e.g. maps multi-omics signals onto KEGG pathway topology to improve interpretability and biomarker discovery (Kanehisa and Goto 2000, Lan *et al.* 2024a). While principled, such prior-constrained architectures operate under a closed-world assumption: they are capable of representing only mechanisms present in current annotations, and may underperform when clinically relevant drivers arise from non-canonical interactions, non-coding regulation, or patient-specific epigenetic programs that are incompletely catalogued.

Transformer models offer a complementary route. Self-attention enables global, content-dependent interaction modelling and has proven effective across domains from language to vision (Vaswani *et al.* 2017). Large language models have more recently been applied to a broad range of biomedical data analysis tasks, including omics-related problems (Lan *et al.* 2025), though such approaches typically operate on tokenized sequences and text rather than structured, high-dimensional molecular feature tables of the kind considered here. However, omics profiles differ fundamentally from sequences and images: features form unordered sets without a natural positional structure, and naïve gene tokenization can violate permutation invariance and distort biological correlations. Set-based attention models—notably the Set Transformer—formalize permutation-invariant processing and provide a principled motivation for embedding strategies that treat omics features as unordered collections (Lee *et al.* 2019). Effective omics Transformers additionally require strong regularization to prevent overfitting; self-supervised reconstruction objectives, including denoising autoencoders and masked autoencoding, offer a principled mechanism to learn robust representations from corrupted inputs (Vincent *et al.* 2008, He *et al.* 2022).

Here we introduce OmicsTransformer, an end-to-end multi-omics framework that couples self-supervised manifold learning with uncertainty-aware fusion. Each modality is first projected into multiple latent patches, and semantic consistency between embeddings computed from full and randomly masked inputs is enforced via an Exponential Cosine Consistency Loss. This objective acts as a structural inductive bias: by compelling the model to reconstruct masked semantics from correlated features, it suppresses independent noise and organizes each modality into a smooth latent manifold that preserves continuous biological gradients. A Transformer encoder then captures higher-order dependencies across patches, and an uncertainty-weighted fusion module integrates modalities according to sample-specific confidence (Gal and Ghahramani 2016, Kendall and Gal 2017). Modality dropout during training, inspired by multimodal dropout strategies (Neverova *et al.* 2016), further enhances robustness to missing assays.

OmicsTransformer is benchmarked on eight cohorts spanning diagnostic subtyping and grading [The Cancer Genome Atlas breast invasive carcinoma (TCGA-BRCA), TCGA lower-grade glioma (TCGA-LGG), and the Religious Orders Study and Memory and Aging Project (ROSMAP)] and prognostic recurrence prediction (TCGA-LIHC, TCGA-BLCA, TCGA-PRAD, TARGET-WT, and a recurrence subset of TCGA-BRCA). Our experiments demonstrate a new state-of-the-art across all tasks, with particularly large gains in challenging prognostic settings, supporting the premise that learning clean, data-driven manifolds can outperform both heuristic topology construction on raw features and pathway-restricted modelling for recurrence prediction.

In summary, our contributions are 3-fold. First, a relation-aware masked consistency learning strategy that denoises high-dimensional omics data and discovers intrinsic manifolds *de novo*, overcoming the limitations of raw-similarity graphs and static biological priors. Second, a Transformer-based global modelling pipeline adapted to unordered omics embeddings, enabling extraction of globally contextualized representations that reveal continuous biological gradients. Third, an uncertainty-weighted fusion mechanism with modality dropout that improves robustness to missing modalities and supports biomarker discovery through attribution-based interpretation.

## 2 Materials and methods

### 2.1 The OmicsTransformer framework

**Figure 1:**
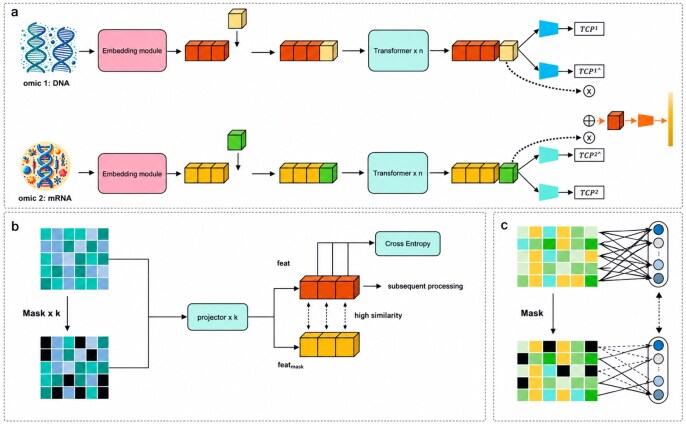
Overview of the OmicsTransformer framework. (a) System Architecture: The pipeline comprises three hierarchical stages: (i) Relation-Aware Embedding, which projects high-dimensional unordered features into latent subspaces (‘patches’); (ii) Global Context Encoding, utilizing a Transformer encoder to capture high-order interactions; and (iii) Uncertainty-Weighted Fusion, which dynamically integrates modalities based on predictive confidence (True Class Probability) to ensure robustness against incomplete data. (b) Masked-Loss Mechanism: Illustration of the self-supervised Exponential Cosine Consistency Loss. By minimizing the angular distance between embeddings of original and partially masked inputs, the model exploits biological redundancy to enforce semantic invariance. (c) Manifold Learning: The masked loss acts as a structural inductive bias. It forces the reconstruction of semantic context from correlated biomolecules, effectively filtering out stochastic noise and compressing disordered data into organized, low-dimensional manifolds.

OmicsTransformer is an end-to-end multi-omics learning framework designed for (i) diagnostic stratification (subtyping/grading) and (ii) prognostic recurrence prediction. The core methodological goal is to learn robust, biologically meaningful patient representations directly from high-dimensional, redundant and partially missing omics measurements, while retaining interpretability through stable biomarker attribution (Fig. 1).

### 2.2 Problem definition

We formulate multi-omics analysis as two supervised learning problems: (i) disease diagnosis via multi-class subtyping and (ii) prognostic recurrence prediction as a binary classification task.

Let the dataset be denoted as $D={\left\{\left(x_{i},\;y_{i}\right)\right\}}_{i=1}^{N}$, where *N* is the number of subjects. For the Diagnosis task: $y_{i}\;\in \;\{1,\;\ldots ,\;C\}$ represents discrete disease subtypes. For the Prognosis task: $y_{i}\;\in \;\{0,\;1\}$ represents the clinical outcome (0: Non-recurrence, 1: Tumour Recurrence).

For each subject *i*, the input $X_{i}=\{x_{i}^{\left(1\right)},\;\ldots ,\;x_{i}^{\left(M\right)}\}$ consists of feature vectors from *M* distinct omics modalities (e.g. mRNA, DNA methylation). Here, $x_{i}^{\left(m\right)}\;\in \;R^{D_{m}}$ represents the high-dimensional feature vector of the mth modality with original dimension $D_{m}$. The goal is to learn a mapping function $F\text{:}\;X_{i}\;\to \;y_{i}$.

### 2.3 Cohorts, data sources and data preprocessing

We evaluated OmicsTransformer on eight cohorts spanning three diagnostic benchmarks (TCGA-BRCA subtyping, TCGA-LGG grading, ROSMAP Alzheimer’s disease classification) and five prognostic benchmarks (recurrence prediction in TCGA-LIHC, TCGA-BLCA, TCGA-PRAD, TARGET-WT, and a recurrence subset of TCGA-BRCA). Cohort composition, modalities, and post-selection feature counts are summarized in Table 1 and Table 1, available as supplementary data at *Bioinformatics* online. TCGA cohorts were obtained from The Cancer Genome Atlas program (Weinstein *et al.* 2013). Paediatric Wilms tumour data were obtained from the Therapeutically Applicable Research to Generate Effective Treatments (TARGET) initiative (Ma *et al.* 2018). ROSMAP data were obtained from the Religious Orders Study and Memory and Aging Project and the associated multi-omic atlas (Bennett *et al.* 2012, De Jager *et al.* 2018).

Preprocessing was applied per modality and per cohort, following standard practice in multi-omics classification pipelines (Wang *et al.* 2021a, Ouyang *et al.* 2023, Zhao *et al.* 2024). Raw TCGA per-modality dimensionality—∼60 000 mRNA genes, ∼485 000 DNA methylation CpG sites, and ∼1900 mature miRNAs—far exceeds cohort size, necessitating dimensionality reduction. For each diagnostic cohort, missing values were imputed by zero-fill and near-zero-variance features removed. We then applied one-way ANOVA within each modality, using the F-statistic purely as a ranking criterion and retaining a fixed top-k set of features; k was chosen to match the feature budgets used by baseline methods on the same cohorts [e.g. 1000 mRNA/1000 methylation/500 miRNA for TCGA-BRCA, following Wang *et al.* (2021a); no *P*value threshold was imposed]. This ensures OmicsTransformer and all baselines are evaluated under an identical feature budget per modality; post-selection dimensions are listed in Table 1. Retained features were log-transformed (log_2_(X + 1)) and Min–Max normalized to [0, 1].

For prognostic tasks, we used a biologically guided feature selection strategy to facilitate fair comparison with pathway-informed baselines (Lan *et al.* 2024a). For mRNA and miRNA, we retained features annotated in KEGG (Kanehisa and Goto 2000) after removing non-coding genes, and applied min-max normalization. For somatic mutation (SNV) data, each gene was binarized (1 if any qualifying variant was observed in that gene; 0 otherwise).

### 2.4 OmicsTransformer architecture

Unlike images with grid structure, omics profiles are high-dimensional, sparse, and permutation-invariant. To mitigate the curse of dimensionality and render unordered omics features amenable to Transformer processing, we introduce a masked-loss-guided embedding module. This module maps raw features into multiple latent subspaces (‘patches’) while explicitly encouraging preservation of intrinsic biomolecular correlations.

#### 2.4.1 Relation-aware patch embedding

Each modality is treated as an unordered feature set. For each modality m, we initialize K independent linear projectors to map the input $x_{i}^{\left(m\right)}$ into *K* distinct low-dimensional embedding vectors, each parameterized by a weight matrix $W_{k}^{\left(m\right)}\;\in \;R^{d\times D_{m}}$ and a bias vector $b_{k}^{\left(m\right)}\in \;R^{d}$. The kth embedding token $v_{i,k}^{\left(m\right)}\;\in \;R^{d}$ is computed as:


(1) $$v_{i,\;k}^{\left(m\right)}=\text{ELU}(W_{k}^{\left(m\right)}x_{i}^{(m)\;}+b_{k}^{(m)\;}),\;(k=1,\;\ldots ,\;K)$$

Here, d is the dimension of the embedding space. The Exponential Linear Unit (ELU) activation is used to perform non-linear transformation while mitigating the vanishing gradient problem, allowing the model to robustly handle negative values. This yields a sequence of embeddings $V_{i,\;k}^{\left(m\right)}=[v_{i\;,\;1}^{\left(m\right)},\ldots ,\;v_{i,\;K}^{\left(m\right)}]$, representing the sample across multiple latent manifolds.

#### 2.4.2 Masked consistency regularization

To prevent overfitting and force the model to learn intrinsic molecular correlations (e.g. gene co-expression networks), we use a self-supervised masked learning strategy. During training, we generate a masked input: ${\tilde{x}}_{i}^{\left(m\right)}=x_{i}^{\left(m\right)}⊙M$, where $M\;\in {\left\{0,1\right\}}^{D_{m}}\;$is a binary mask vector sampled from a Bernoulli distribution with parameter *r* (masking rate). The same *K* projectors are applied to both original input $x_{i}^{\left(m\right)}$ and the masked input ${\tilde{x}}_{i}^{\left(m\right)}$ to obtain embeddings $v_{i,\;k}^{\left(m\right)}$ and ${\tilde{v}}_{i,\;k}^{\left(m\right)}$, respectively. We define the Exponential Cosine Similarity Loss ($L_{\text{mask}}$) to maximize the consistency:


(2) $$L_{\text{mask}}=\frac{1}{N}\sum_{i=1}^{N}\sum_{k=1}^{K}\left(e‐\;\text{exp}\;(\frac{v_{i,k}^{\left(m\right)}\cdot {\tilde{v}}_{i,k}^{\left(m\right)}}{|\left|v_{i,k}^{\left(m\right)}\right||\left|{\tilde{v}}_{i,k}^{\left(m\right)}\right|})\right)$$

This objective penalizes angular discrepancies between the masked and unmasked representations. The exponential term increases the penalty when similarity is high, thereby encouraging reconstruction of masked semantics from correlated unmasked features. In this way, the loss provides a structural inductive bias that suppresses independent stochastic noise while retaining correlated biological signals.

#### 2.4.3 Patch-level auxiliary supervision

To ensure the learned subspaces retain discriminative information for the classification task, we attach a lightweight linear classifier to each embedding patch. The auxiliary task loss $L_{\text{task}}$ is defined as the cross-entropy loss of all patches:


(3) $$L_{\text{task}}=-\frac{1}{N}\sum_{i=1}^{N}\sum_{k=1}^{K}\;\text{log}\;p(y_{i}|v_{i,\;k}^{\left(m\right)})$$

where $p(y_{i}|v_{i,\;k}^{\left(m\right)})$ is the probability of the true label predicted by the kth patch. The total embedding loss is $L_{\text{emb}}=\lambda_{1}L_{\text{mask}}+\lambda_{2}L_{\text{task}}$. In this study, we set $\lambda_{1}=\lambda_{2}=1$.

#### 2.4.4 Transformer encoder for global context

To model long-range dependencies across the K embedding patches, we use a standard Transformer Encoder. A learnable classification token, [CLS], is prepended to the sequence $V_{i,\;k}^{\left(m\right)}=[v_{i\;,\;1}^{\left(m\right)},\;\ldots ,\;v_{i,\;K}^{\left(m\right)}]$, resulting in an input sequence of length K+1. The encoder consists of L layers. Each layer contains Multi-Head Self-Attention (MSA) and a Feed-Forward Network (FFN). The Self-Attention mechanism computes the relevance between patches. Given query Q, key k, and value V matrices, where $Q,\;K,\;V\;\in \;R^{N\times d_{m}}$, the self-attention is applied through a scaled dot-product:


(4) $$\text{Attention}(Q,\;K,\;V)=\text{Softmax}(\frac{QK^{T}}{\sqrt{d_{m}}})V$$

Through this mechanism, the [CLS] token aggregates global information from all patches. The output vector corresponding to the [CLS] token, denoted as $h_{i}^{\left(m\right)}\;\in \;R^{d}$, serves as the aggregated global representation for modality *m*.

#### 2.4.5 Uncertainty-weighted multimodal fusion

Traditional concatenation ignores the varying quality of different omics layers. We use an Uncertainty-Weighted Fusion strategy that adaptively weights modalities based on their predictive confidence.

##### 2.4.5.1 True class probability

We quantify the reliability of modality m using its True Class Probability (TCP)—defined as the probability assigned to the ground-truth label $y_{i}$ by the modality-specific classifier. Let $p_{i}^{\left(m\right)}$ be the probability vector predicted by modality m using $h_{i}^{\left(m\right)}$. The TCP is given by ${\text{TCP}}_{i}^{\left(m\right)}=p_{i}^{\left(m\right)}[y_{i}]$. Note that ${\text{TCP}}_{i}^{\left(m\right)}$ reflects the actual correctness of the modality and is only available during training.

##### 2.4.5.2 Confidence estimation and fusion

Since the true label $y_{i}$ is unknown during inference, we train a dedicated Confidence Network (a small MLP) to predict this TCP value. Let ${\hat{\text{TCP}}}_{i}^{\left(m\right)}$ be the estimated confidence score for sample i, modality m. The confidence loss $L_{\text{conf}}$ minimizes the mean squared error between the estimated and the actual TCP, while also supervising the modality-specific prediction:


(5) $$L_{\text{conf}}=\frac{1}{N}\sum_{i=1}^{N}\sum_{m=1}^{M}\left[{\left({\text{TCP}}_{i}^{\left(m\right)}-{\hat{\text{TCP}}}_{i}^{\left(m\right)}\right)}^{2}+L_{\text{CE}}(p_{i}^{\left(m\right)},\;y_{i})\right]$$

where $L_{\text{CE}}\;$is the cross-entropy loss for the modality-specific prediction.

##### 2.4.5.3 Weighted fusion

The final fused representation $h_{i}^{\text{final}}$ is obtained by weighting each modality’s representation by its estimated confidence:


(6) $$h_{i}^{\text{final}}=\sum_{m=1}^{M}{\hat{\text{TCP}}}_{i}^{(m)\;}\cdot \;h_{i}^{\left(m\right)}$$

Finally, a classifier transforms $h_{i}^{\text{final}}$ into the final class probabilities, optimized via cross-entropy loss $L_{\text{pred}}^{\text{final}}$, where $L_{\text{pred}}^{\text{final}}=-\sum_{k=1}^{K}y_{k}\;\text{log}\;{\;p}_{k}$. Then we can get the total objective loss function:


(7) $$L_{\text{total}}=\sum_{m=1}^{M}(L_{\text{emb}}^{\left(m\right)}+L_{\text{conf}}^{\left(m\right)})+L_{\text{pred}}^{\text{final}}$$

Optimizing the total loss $L_{\text{total}}$ yields the final model.

### 2.5 Modality dropout for incomplete assays

To simulate clinical settings where some assays are unavailable, we apply modality dropout during training: with a specified probability, an entire modality is removed for a sample (its tokens are zeroed and excluded from fusion). This training strategy is inspired by ModDrop and related multimodal dropout methods (Neverova *et al.* 2016) and improves robustness under test-time missing modalities.

### 2.6 Implementation details

Models were implemented in PyTorch and optimized using Adam (Kingma and Ba 2015). For each cohort, architectural depth and embedding dimension were tuned to balance expressivity and overfitting risk (Table 2, available as supplementary data at *Bioinformatics* online). Because several cohorts have limited sample size, we set the mini-batch size to one fifth of each cohort’s training-set size (i.e. five mini-batches per epoch) in order to avoid statistically unstable batches; inference uses the same batch size. Unless otherwise noted, all reported results are aggregated over 20 independent training runs with different random seeds and report mean ± standard deviation for Accuracy (ACC) and Weighted F1 (WF1), together with Macro F1 for multi-class tasks (BRCA subtyping) or area under the receiver operating characteristic curve (AUC) for binary tasks (LGG/ROSMAP and all recurrence tasks).

**Table 2:** TCGA-BRCA PAM50 subtyping (five-class).^a^

	BRCA		
Method	ACC	WeightedF1	MacroF1
SVM	82.40%±3.3	82.79%±3.3	79.81%±4.1
KNN	74.22%±0.5	70.83%±0.5	65.62%±0.6
RF	75.29%±0.7	73.70%±0.8	65.02%±1.6
NN	79.31%±3.3	77.98%±4.7	71.82%±7.3
LR	80.15%±0.6	80.05%±0.8	75.08%±0.8
MOGONET	78.51%±1.8	76.53%±2.4	69.66%±2.6
MOGLAM	85.33%±0.7	85.90%±0.7	81.81%±0.7
CLCLSA	79.47%±2.4	79.07%±2.5	72.29%±2.8
MMdynamic	86.73%±0.7	86.98%±0.8	82.86%±1.1
OmicsTransformer	**89.43%±0.4**	**89.74%±0.4**	**86.33%±0.8**

a Comparison of OmicsTransformer with classical machine-learning baselines and representative deep multi-omics integration methods. Metrics include accuracy (ACC), weighted F1 (WF1), and macro F1 (MacroF1). Values are mean ± standard deviation across 20 independent runs; best results are highlighted in bold.

#### 2.6.1 Computational complexity and scaling

OmicsTransformer’s three principal components exhibit distinct scaling regimes. (i) The embedding layer applies K linear projectors of size *d* × *D*, incurring per-sample cost *O*(*K*·*d*·*D*)—linear in *D*. (ii) The Transformer encoder operates over only *K* + 1 tokens and is independent of *D*, with per-sample cost *O*(*L*·(*K* + 1)^2^·*d*). (iii) Fusion scales only with the number of modalities *M* and with *d*. Training cost is therefore linear in both cohort size *N* and feature dimension *D*, with no pairwise patient-similarity computation; this contrasts with graph-based pipelines whose graph-construction cost is *O*(*N*^2^·*D*) per modality. By restricting self-attention to a small fixed set of latent patches, the architecture remains practical for *D* > 10^4^–10^5^ and cohorts of *N* ≥ 10^5^ without prohibitive memory growth.

#### 2.6.2 Empirical computational cost

Training and inference cost on the cohorts was measured on a single NVIDIA RTX 4070ti (20 seeds, mixed precision; Table 3, available as supplementary data at *Bioinformatics* online). Inference time is reported per test split at the training batch size (one fifth of the training-set size), and reflects GPU compute only, excluding one-time data loading and preprocessing. Training is tractable on commodity GPU hardware, consistent with the linear-in-*D*, linear-in-*N* scaling above.

**Table 3:** TCGA-LGG grading (Grade II versus Grade III).^a^

	LGG		
Method	ACC	WeightedF1	AUC
SVM	83.79%±0.3	83.72%±0.3	90.16%±0.1
KNN	78.43%±3.2	78.37%±3.5	85.89%±3.6
RF	78.89%±2.4	78.87%±2.5	88.09%±0.8
NN	76.76%±9.5	74.79%±14.3	86.30%±11.5
LR	81.86%±0.3	81.86%±0.3	89.16%±0.1
MOGONET	76.91%±1.5	76.73%±7.7	86.44%±0.9
MOGLAM	84.97%±0.1	84.97%±0.1	87.50%±0.1
CLCLSA	80.82%±1.0	80.79%±1.1	86.26%±0.8
MMdynamic	83.59%±1.0	83.55%±1.3	89.17%±0.5
OmicsTransformer	**85.29%±0.7**	**85.28%±0.7**	**90.59%±0.3**

a Diagnostic performance comparison across baselines. Metrics include accuracy (ACC), weighted F1 (WF1), and area under the ROC curve (AUC). Values are mean ± standard deviation across 20 independent runs; best results are highlighted in bold.

### 2.7 Baselines

We compared OmicsTransformer to (i) classical machine-learning methods (KNN, SVM, Random Forest, logistic regression, and a shallow neural network) implemented with scikit-learn (Pedregosa *et al.* 2011), and (ii) representative deep multi-omics integration baselines: MOGONET (Wang *et al.* 2021a), MOGLAM (Ouyang *et al.* 2023), CLCLSA (Zhao *et al.* 2024), MMdynamic (Han *et al.* 2022), and the pathway-informed DeepKEGG framework (Lan *et al.* 2024a) using KEGG pathway annotations (Kanehisa and Goto 2000). Baseline hyperparameters were tuned under the same evaluation protocol (Table 4, available as supplementary data at *Bioinformatics* online).

**Table 4:** ROSMAP Alzheimer’s disease classification (AD versus control).^a^

	ROSMAP		
Method	ACC	WeightedF1	AUC
SVM	79.20%±0.2	79.14%±0.2	86.35%±0.1
KNN	74.18%±0.4	70.79%±0.4	84.71%±0.4
RF	80.33%±2.3	80.33%±2.3	87.39%±1.7
NN	78.11%±2.3	77.78%±2.5	87.22%±0.8
LR	81.13%±0.1	81.14%±0.1	88.43%±0.1
MOGONET	78.94%±1.5	79.83%±1.5	87.61%±1.3
MOGLAM	77.02%±2.0	77.02%±2.0	81.81%±0.7
CLCLSA	79.47%±2.4	79.07%±2.5	85.12%±1.2
MMdynamic	82.07%±2.0	82.78%±1.9	**89.58%±1.3**
OmicsTransformer	**85.66%±1.2**	**85.66%±1.2**	89.24%±0.5

a Comparison of OmicsTransformer with baselines using accuracy (ACC), weighted F1 (WF1), and AUC. Values are mean ± standard deviation across 20 independent runs; best results are highlighted in bold.

### 2.8 Evaluation protocol

Performance was evaluated using accuracy (ACC), weighted F1 (WF1), and either macro-F1 (multi-class diagnosis) or area under the receiver operating characteristic curve (AUC; binary diagnosis/prognosis). We report mean ± standard deviation across 20 independent runs. Receiver operating characteristic (ROC) curves were computed from the aggregated prediction scores. For qualitative visualization of representation geometry, we used t-SNE (van der Maaten and Hinton 2008).

### 2.9 Biomarker attribution and ensemble stability

To identify candidate biomarkers, we estimate feature importance at the embedding stage rather than relying on attention weights, which are not guaranteed to provide faithful explanations (Jain and Wallace 2019). To accurately quantify feature contribution, we propose a Variance-Weighted Activation Attribution metric. Let $W_{k}^{\left(m\right)}\;\in \;R^{d\times D_{m}}$ denote the weight matrix for the kth latent patch of modality m, and $\sigma_{j}^{2}$ be the variance of the activation output of the jth latent dimension across the cohort. This variance proxies the discriminative power of that dimension. The effective importance score $S_{i,\;j}$ for the ith input molecule is defined as the weight magnitude modulated by the discriminative power of its corresponding latent dimension:


(8) $$S_{i,j}=\sum_{j=1}^{d}|W_{k,\;i,\;j}^{\left(m\right)}|\cdot \sigma_{j}^{2}$$

For each patch, we extract the top-20 features with the highest $S_{i,\;j}$ to form a candidate molecular set.

For each patch, we select the top-ranked features to form a patch-level candidate set. To improve reproducibility, we train 20 independent models and aggregate patch-level sets using ensemble stability selection (Meinshausen and Bühlmann 2010). We report (i) Robust Gradient Drivers obtained by union aggregation after patch alignment across runs and (ii) the Minimum Essential Core obtained by strict intersection across aligned patches (Tables 5–8, available as supplementary data at *Bioinformatics* online).

**Table 5:** Prognostic recurrence prediction across five cohorts.^a^

Dataset	Metric	Omics-transformer	DeepKEGG	MMdynamic	MOGONET	MOGLAM	CLCLSA
**LIHC**	**ACC**	**98.32 ± 1.17**	76.82 ± 0.98	69.27 ± 4.71	76.01 ± 1.40	75.70 ± 2.10	72.90 ± 2.10
	**WF1**	**98.32 ± 1.17**	76.72 ± 0.96	67.36 ± 5.69	76.48 ± 1.50	76.11 ± 1.91	72.89 ± 2.10
	**AUC**	**99.88 ± 0.18**	91.51 ± 0.39	75.10 ± 7.39	80.88 ± 1.60	82.70 ± 1.62	74.78 ± 3.00
**BLCA**	**ACC**	**92.93 ± 0.80**	81.82 ± 0.64	71.24 ± 5.44	71.32 ± 1.60	75.74 ± 2.00	71.01 ± 1.10
	**WF1**	**92.92 ± 0.82**	82.01 ± 0.63	54.89 ± 7.69	71.63 ± 1.40	76.12 ± 1.90	69.61 ± 1.00
	**AUC**	**97.22 ± 0.70**	91.05 ± 0.15	74.67 ± 6.67	78.37 ± 1.80	82.75 ± 1.60	67.86 ± 1.10
**BRCA**	**ACC**	**90.39 ± 1.80**	81.02 ± 1.45	85.90 ± 5.71	85.31 ± 2.54	81.22 ± 1.50	80.31 ± 7.20
	**WF1**	**90.20 ± 1.87**	80.90 ± 1.45	84.39 ± 6.07	84.62 ± 2.85	80.27 ± 1.80	73.6 ± 10.90
	**AUC**	**96.23 ± 1.67**	87.25 ± 0.46	90.26 ± 6.30	87.27 ± 3.82	91.43 ± 1.30	74.8 ± 12.62
**WT**	**ACC**	**86.62 ± 2.37**	78.53 ± 2.48	82.26 ± 6.97	79.73 ± 3.80	74.69 ± 3.30	76.47 ± 5.20
	**WF1**	85.75 ± 2.51	79.90 ± 2.10	**88.60 ± 9.22**	78.01 ± 3.10	76.33 ± 2.90	68.82 ± 5.33
	**AUC**	**88.31 ± 2.32**	85.03 ± 0.65	82.65 ± 5.36	76.67 ± 3.00	81.16 ± 2.40	86.24 ± 6.10
**PRAD**	**ACC**	**87.60 ± 2.07**	62.40 ± 1.00	86.34 ± 5.98	69.55 ± 1.50	65.43 ± 2.30	77.07 ± 4.73
	**WF1**	**87.47 ± 2.12**	62.76 ± 0.99	84.32 ± 6.29	69.75 ± 2.60	61.36 ± 3.00	64.1 ± 10.63
	**AUC**	**93.58 ± 0.94**	76.13 ± 0.26	89.60 ± 6.64	74.83 ± 2.60	61.11 ± 2.40	62.6 ± 11.59

a We report accuracy (ACC), weighted F1 (WF1), and AUC for OmicsTransformer and deep multi-omics baselines (including pathway-informed and graph-based models). Values are mean ± standard deviation (%) across 20 independent runs; best results per cohort/metric are highlighted in bold. Modality availability for each cohort follows Table 1.

**Table 6:** Effect of modality dropout under complete-modality evaluation.^a^

Dataset	Method	ACC	WeightedF1	MacroF1/AUC
BRCA	MMdynamic	86.73%±0.7	86.98%±0.8	82.86%±1.1
	MMdynamic-I	88.25%±0.4	88.56%±0.4	84.88%±0.6
	OmicsTransformer	89.18%±0.7	89.48%±0.8	86.04%±1.0
	OmicsTransformer-I	**89.43%±0.4**	**89.74%±0.4**	**86.33%±0.8**
LGG	MMdynamic	83.59%±1.0	83.55%±1.3	89.17%±0.5
	MMdynamic-I	84.38%±0.6	84.37%±0.7	89.32%±0.28
	OmicsTransformer	85.16%±0.9	85.15%±0.9	90.38%±0.4
	OmicsTransformer-I	**85.29%±0.7**	**85.28%±0.7**	**90.59%±0.3**
ROSMAP	MMdynamic	82.07%±2.0	82.78%±1.9	89.58%±1.3
	MMdynamic-I	82.12%±1.2	83.00%±1.1	**89.86%±0.8**
	OmicsTransformer	85.05%±1.4	85.04%±1.4	89.35%±0.6
	OmicsTransformer-I	**85.66%±1.2**	**85.66%±1.2**	89.24%±0.5

a Models are trained either without modality dropout (no suffix) or with modality dropout (‘-I’) and evaluated using all modalities. For BRCA (multi-class), the third metric is MacroF1; for LGG/ROSMAP (binary), the third metric is AUC. Values are mean ± standard deviation across 20 independent runs; best results are highlighted in bold.

**Table 7:** Robustness to missing modalities at inference.^a^

Dataset	Training Regime	ACC	WeightedF1	MacroF1/AUC
BRCA	Dropout Training	**83.16%±0.6**	**82.75%±0.6**	**78.45%±0.8**
	Complete Training	76.40%±1.8	75.90%±2.0	69.23%±3.3
LGG	Dropout Training	**82.28%±1.0**	**82.21%±1.0**	**88.11%±0.7**
	Complete Training	71.04%±1.6	70.21%±2.1	79.37%±1.4
ROSMAP	Dropout Training	**76.62%±1.2**	**76.57%±1.2**	**84.56%±0.6**
	Complete Training	69.92%±1.9	68.47%±2.5	71.99%±0.9

a Models are trained with modality dropout (Dropout Training) or without (Complete Training) and evaluated after randomly removing one modality at test time. For BRCA (multi-class), the third metric is MacroF1; for LGG/ROSMAP (binary), the third metric is AUC. Values are mean ± standard deviation across 20 independent runs; best results are highlighted in bold.

**Table 8:** Ablation study of key components.^a^

Dataset	Method	ACC	WeightedF1	MacroF1/AUC
BRCA	NO MASK	87.17%±0.7	87.43%±0.8	83.43%±0.9
	INTEGRATION NOT	88.14%±0.6	88.47%±0.6	85.46%±0.8
	SPLIT	87.22%±0.9	87.76%±1.0	84.25%±1.5
	Proposed	**89.18%±0.7**	**89.48%±0.8**	**86.04%±1.0**
LGG	NO MASK	81.31%±0.9	81.27%±0.9	89.76%±0.6
	INTEGRATION NOT	83.01%±1.5	83.00%±1.5	89.00%±0.5
	SPLIT	82.91%±1.2	82.80%±1.2	88.68%±0.6
	Proposed	**85.16%±0.9**	**85.15%±0.9**	**90.38%±0.4**
ROSMAP	NO MASK	80.52%±3.1	80.52%±3.1	87.56%±1.9
	INTEGRATION NOT	83.77%±1.6	83.76%±1.6	88.99%±0.5
	SPLIT	81.13%±2.7	81.07%±2.7	87.54%±1.2
	Proposed	**85.05%±1.4**	**85.04%±1.4**	**89.35%±0.6**

a NO MASK: training without the Exponential Cosine Consistency Loss; INTEGRATION NOT: replacing uncertainty-weighted fusion with simple averaging; SPLIT: naive feature partitioning without relation-aware embedding. Metrics follow Tables 2–4 (MacroF1 for BRCA; AUC for LGG/ROSMAP). Values are mean ± standard deviation across 20 independent runs; best results are highlighted in bold.

## 3 Results

### 3.1 Overview of OmicsTransformer

Figure 1 illustrates OmicsTransformer as a three-stage pipeline for multi-omics prediction. The model first constructs relation-aware embeddings by projecting each modality into multiple latent patch tokens under a masked consistency objective; these tokens are subsequently contextualized by a Transformer encoder; and finally, an uncertainty-weighted fusion module integrates modality-specific representations using sample-level confidence estimates (TCP) to mitigate modality imbalance and accommodate missing assays (Kendall and Gal 2017, Vaswani *et al.* 2017).

A central design choice is to learn topology in latent space rather than impose it directly from raw pairwise similarities. Across all cohorts studied, raw omics features are highly redundant—mean cosine similarity consistently exceeds 96%–99% (Table 1, available as supplementary data at *Bioinformatics* online)—a regime in which similarity-based graph construction yields topologically noisy, poorly discriminative structures (Aggarwal *et al.* 2001). OmicsTransformer counteracts this by enforcing semantic invariance between full and randomly masked inputs, thereby denoising hyper-similar profiles and promoting the emergence of smooth continuous manifolds that preserve latent biological gradients.

### 3.2 Superior performance in disease diagnosis and subtyping

**Figure 2:**
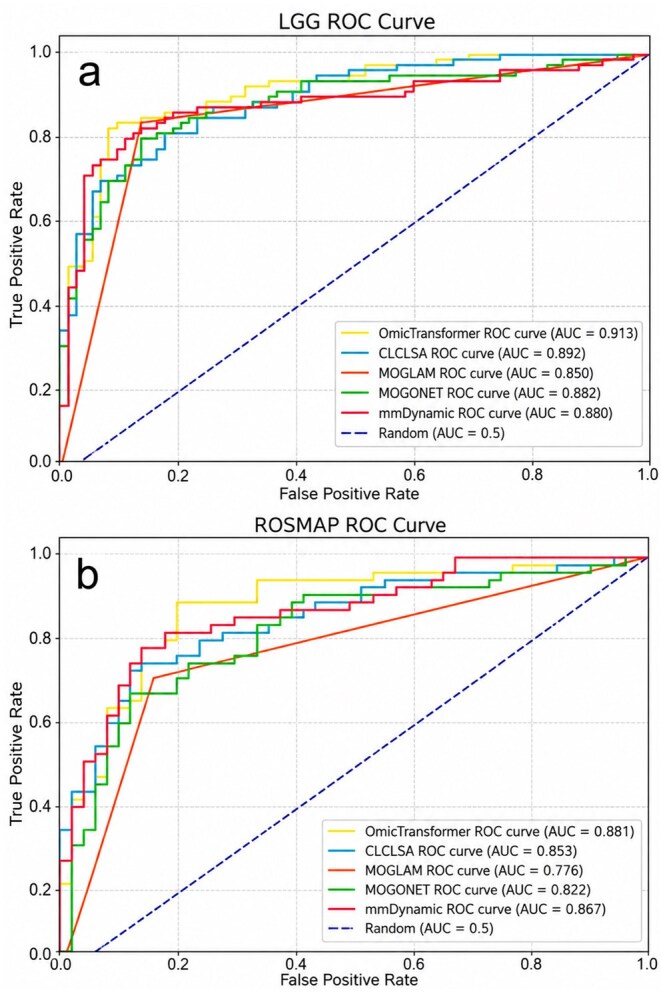
ROC curves for diagnostic benchmarks. Receiver operating characteristic curves comparing OmicsTransformer with baseline methods on (a) TCGA-LGG (Grade II versus Grade III) and (b) ROSMAP (AD versus control). AUC values are reported in the legends; the diagonal line indicates random performance (AUC = 0.5).

Across all three diagnostic benchmarks, OmicsTransformer achieved the best overall performance profile in terms of both mean accuracy and run-to-run stability (Tables 2–4; Fig. 2).

*TCGA-BRCA (complex subtyping)*. On the five-class PAM50 task, OmicsTransformer reached 89.43% ± 0.40% accuracy, with weighted F1 = 89.74% ± 0.40% and macro F1 = 86.33% ± 0.80% (Table 2). This exceeds MMdynamic (86.73% ± 0.70%) and all other baselines, indicating that relation-aware embedding construction improves separability in the presence of complex inter-subtype heterogeneity.

*TCGA-LGG (noisy binary classification)*. For glioma grading, OmicsTransformer achieved 85.29% ± 0.70% accuracy with AUC = 90.59% ± 0.30% (Table 3). The AUC gain over MMdynamic (89.17% ± 0.50%) and graph-based methods such as MOGONET (86.44% ± 0.90%) suggests that masked consistency regularization is particularly beneficial when features are sparse and noisy, and that data-driven topology can be more reliable than raw-similarity graphs under such conditions.

*ROSMAP (small-sample regime)*. Despite the modest cohort size (N = 351), OmicsTransformer maintained 85.66% ± 1.20% accuracy and AUC = 89.24% ± 0.50% (Table 4). Whereas MMdynamic attained a comparable mean AUC (89.58% ± 1.30%), OmicsTransformer improved accuracy by approximately 3.6 percentage points with markedly lower variance, supporting a regularizing effect of masked consistency learning in data-limited settings.

*ROC analysis*. As shown in Fig. 2, OmicsTransformer (yellow curves) achieved the highest AUC across all diagnostic tasks, reaching 0.913 on TCGA-LGG and 0.881 on ROSMAP, confirming consistently superior sensitivity and specificity.

### 3.3 Breakthrough in prognostic recurrence prediction

**Figure 3:**
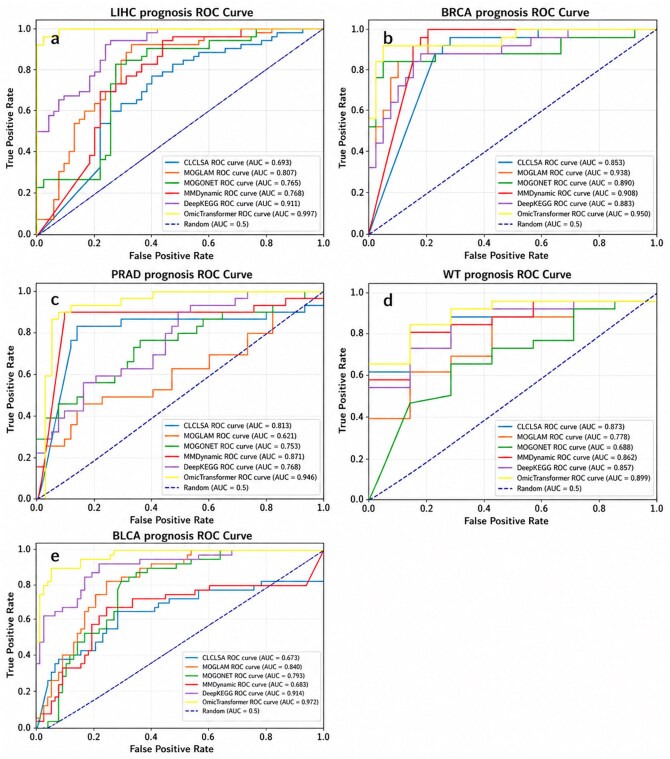
ROC curves for prognostic recurrence prediction. ROC curves for OmicsTransformer and baseline methods on five cohorts: (a) TCGA-LIHC, (b) TCGA-BRCA (recurrence), (c) TCGA-PRAD, (d) TARGET-WT, and (e) TCGA-BLCA. AUC values are reported in the legends; the diagonal line indicates random performance (AUC = 0.5).

Recurrence prediction is inherently more challenging than diagnostic classification: signals are sparser, more patient-specific, and more readily confounded by measurement noise. Across all five prognostic cohorts, OmicsTransformer consistently achieved the best or near-best accuracy, weighted F1, and AUC, with substantially improved run-to-run stability relative to competing deep baselines (Table 5; Fig. 3).

On TCGA-LIHC, OmicsTransformer attained 98.32% ± 1.17% accuracy with a near-saturated AUC of 99.88% ± 0.18%, substantially exceeding DeepKEGG (76.82% ± 0.98%) and MOGONET (76.01% ± 1.40%). On TCGA-BLCA, it achieved 92.93% ± 0.80% accuracy and AUC = 97.22% ± 0.70%, surpassing DeepKEGG by over 11 percentage points (81.82% ± 0.64%); graph-based methods performed poorly on this dataset, with MOGONET reaching only 71.32% ± 1.60%. These results indicate that learning prognostic structure *de novo* can substantially outperform architectures constrained to curated pathway graphs, particularly when clinically relevant recurrence mechanisms extend beyond current pathway annotations (Kanehisa and Goto 2000, Lan *et al.* 2024a). On TCGA-BRCA recurrence, OmicsTransformer reached 90.39% ± 1.80% accuracy and AUC = 96.23% ± 1.67%, improving over DeepKEGG (81.02% ± 1.45%) and providing greater stability than MMdynamic (85.90% ± 5.71%). On TCGA-PRAD, OmicsTransformer achieved 87.60% ± 2.07% accuracy and AUC = 93.58% ± 0.94%, modestly exceeding MMdynamic accuracy (86.34% ± 5.98%) while substantially outperforming DeepKEGG, which degraded to 62.40% ± 1.00%. On TARGET-WT, OmicsTransformer achieved 86.62% ± 2.37% accuracy, exceeding MMdynamic (82.26% ± 6.97%) with markedly lower variance, consistent with more reliable convergence across repeated runs.

*ROC-based evaluation.* ROC curves across prognostic cohorts (Fig. 3) support consistently high discrimination; the figure reports peak AUC values reaching 0.997 (LIHC) and 0.972 (BLCA) for OmicsTransformer.

### 3.4 Robustness to incomplete modalities

To address the clinical reality of missing assays, we compared standard training against modality dropout training under two evaluation regimes. Under complete-modality conditions, dropout provided a modest regularization benefit (Table 6): e.g. OmicsTransformer accuracy improved from 89.18% ± 0.70% to 89.43% ± 0.40% on BRCA and from 85.16% ± 0.90% to 85.29% ± 0.70% on LGG. Under simulated missing-modality inference, dropout-trained models were markedly more resilient: on BRCA, the dropout-trained model achieved 83.16% ± 0.60% versus 76.40% ± 1.80% for the standard model (Table 7). In conjunction with the uncertainty-weighted fusion module, modality dropout training reduces dependence on any single omic source and reliably improves robustness under heterogeneous assay coverage.

### 3.5 Contribution of individual and combined omics modalities

**Figure 4:**
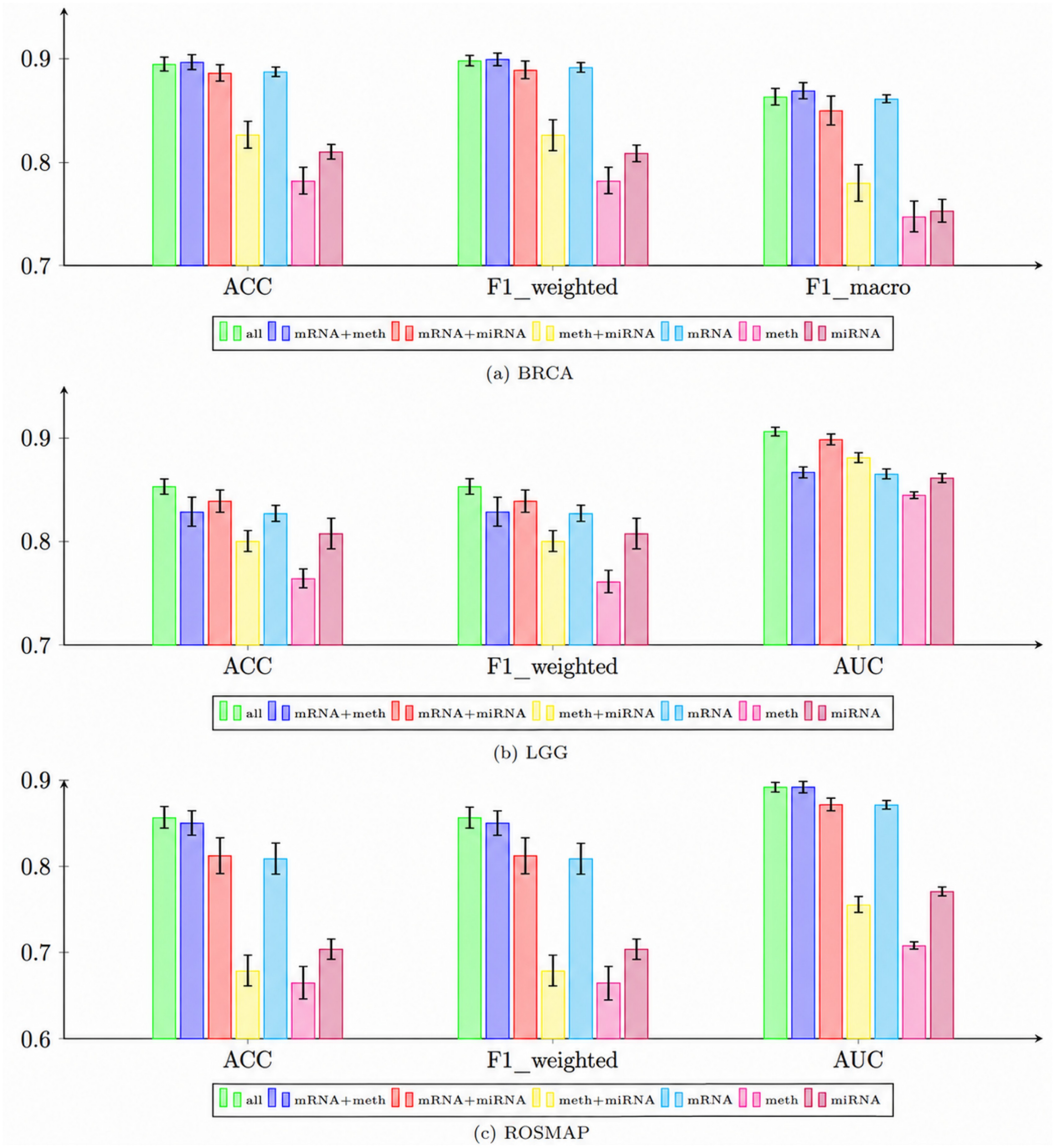
Diagnostic performance under different modality combinations. Bar plots summarize OmicsTransformer performance on (a) BRCA subtyping, (b) LGG grading, and (c) ROSMAP AD classification using all modalities, each pairwise modality combination, and each single modality. Bars show mean across 20 runs and error bars denote ±1 standard deviation. Metrics are ACC, weighted F1, and MacroF1 (BRCA) or AUC (LGG/ROSMAP). ‘meth’ denotes DNA methylation.

**Figure 5:**
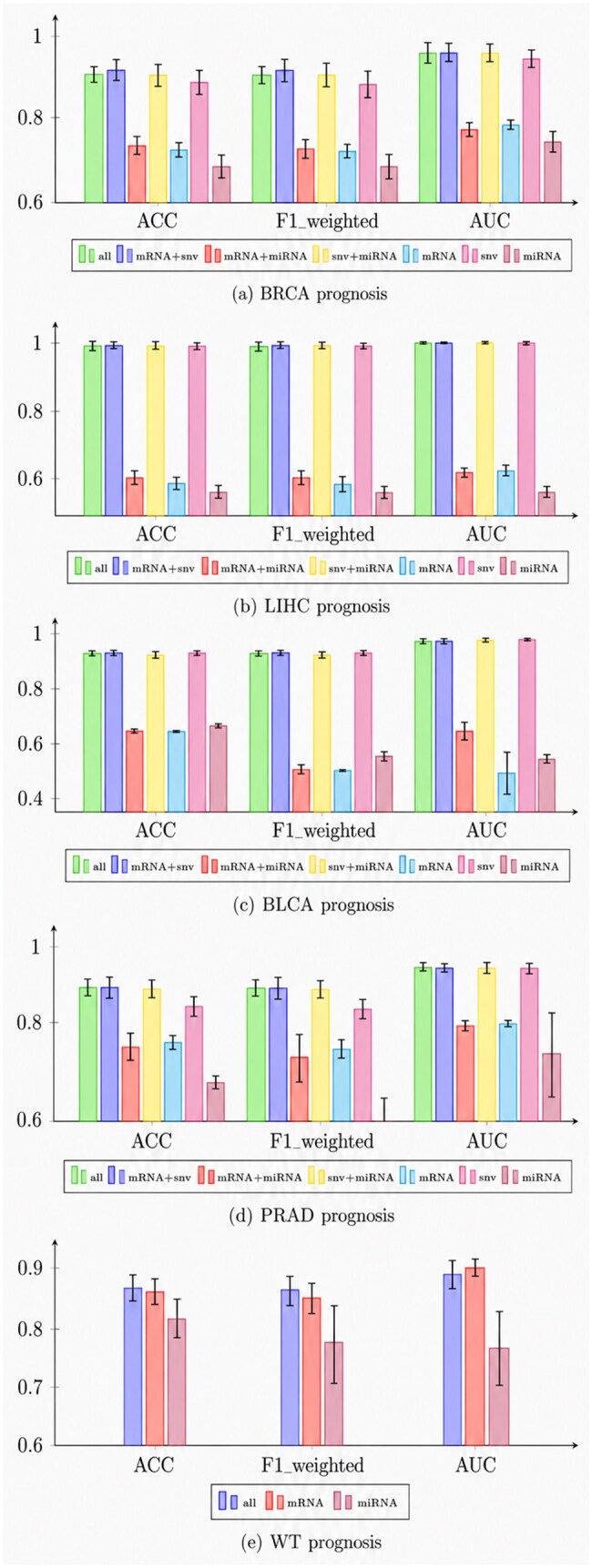
Prognostic performance under different modality combinations. Bar plots summarize OmicsTransformer recurrence prediction performance on (a) BRCA, (b) LIHC, (c) BLCA, (d) PRAD, and (e) WT using all available modalities, pairwise combinations, and single modalities. Bars show mean across 20 runs and error bars denote ±1 standard deviation. Metrics are ACC, weighted F1, and AUC. ‘SNV’ denotes somatic mutation features.

We dissected modality contributions by training OmicsTransformer on single modalities and selected pairwise combinations (Figs 4 and 5). Two consistent patterns emerged across tasks.

*Diagnostic tasks emphasize regulatory layers*. Diagnostic subtyping relied heavily on regulatory information. In BRCA, the combination of mRNA and DNA methylation approached full multi-omics performance, consistent with subtype identity being reflected in transcriptional programs and their epigenetic regulation. In LGG, miRNA emerged as a particularly informative single modality, in line with the well-documented dysregulation of miRNA in glioma.

*Prognostic tasks emphasize genomic instability, with cohort-specific exceptions*. In adult cancer recurrence cohorts (LIHC, BLCA, PRAD, and BRCA recurrence), somatic nucleotide variant (SNV) features were consistently the strongest single modality and often approached the performance of the full multi-omics model. This suggests that genomic instability is the primary recurrence driver in these cancers, while regulatory and transcriptomic layers contribute complementary but secondary signal.

### 3.6 Ablation and sensitivity analyses

Ablation results confirm that the Exponential Cosine Consistency Loss is the principal contributor to performance, with uncertainty-weighted fusion providing additional incremental gains (Table 8). Removing the masked consistency objective (NO MASK) produced the largest degradation—BRCA: 89.18% → 87.17%; LGG: 85.16% → 81.31%—supporting its essential role in denoising and latent space structuring. Replacing uncertainty weighting with simple averaging (INTEGRATION NOT) yielded a consistent 1%–2% drop, while naive feature partitioning (SPLIT) also underperformed the full model, validating the necessity of relation-aware embedding construction over uninformed feature partitioning.

Sensitivity analyses revealed that moderate masking rates (r ≈ 30%–50%) and an embedding subspace count of K ≈ 9–10 yield optimal performance (Figs 1 and 2, available as supplementary data at *Bioinformatics* online), plausibly corresponding to the intrinsic dimensionality of the underlying biological manifolds. This pattern is consistent with a balance between sufficient regularization and retention of enough semantic context to support discriminative downstream learning.

### 3.7 Representation geometry: from noise to manifold

**Figure 6:**
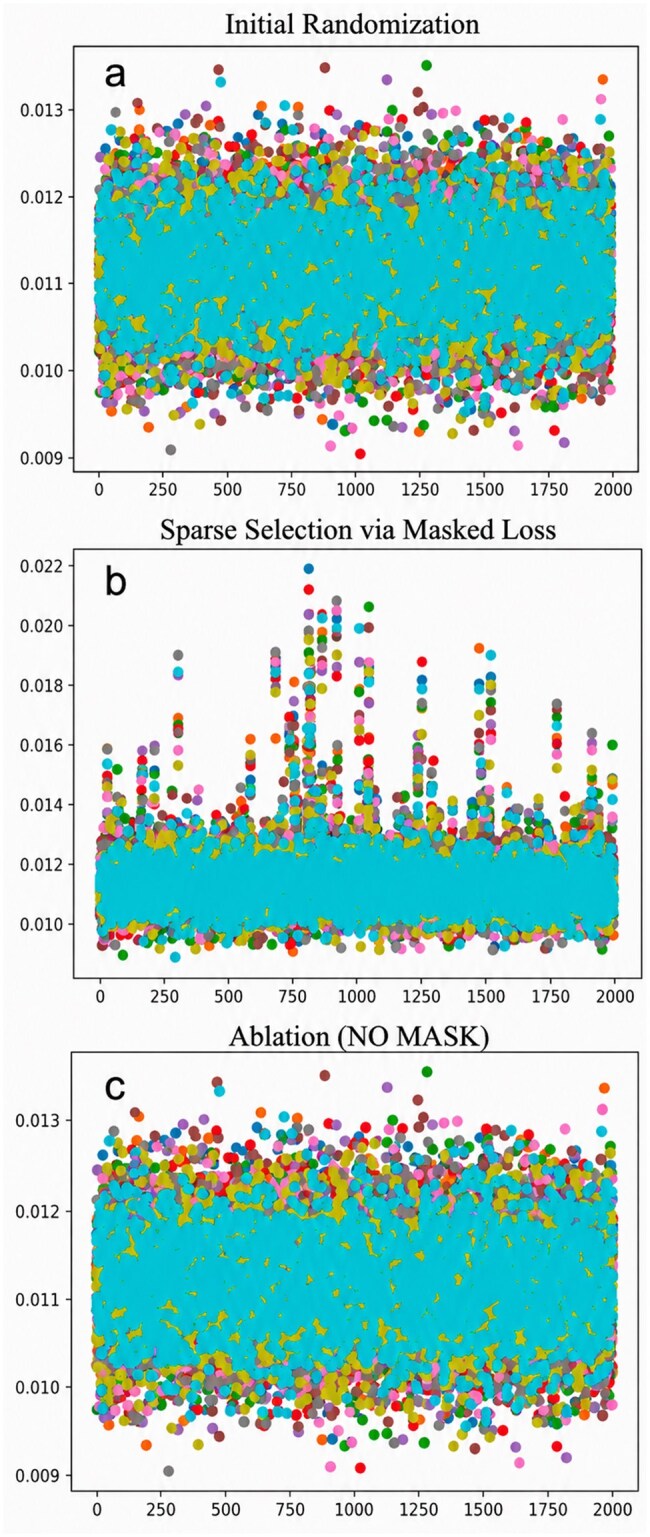
Masked consistency induces sparse, discriminative projection weights in relation-aware embedding. Scatter plots show absolute projection weight magnitudes for input features in a representative embedding projector (same scaling across panels). (a) Random initialization. (b) After training with the masked consistency objective, weight mass concentrates on a small subset of features, indicating implicit feature selection and noise suppression. (c) Ablation without masked consistency (NO MASK) yields a diffuse distribution similar to initialization.

**Figure 7:**
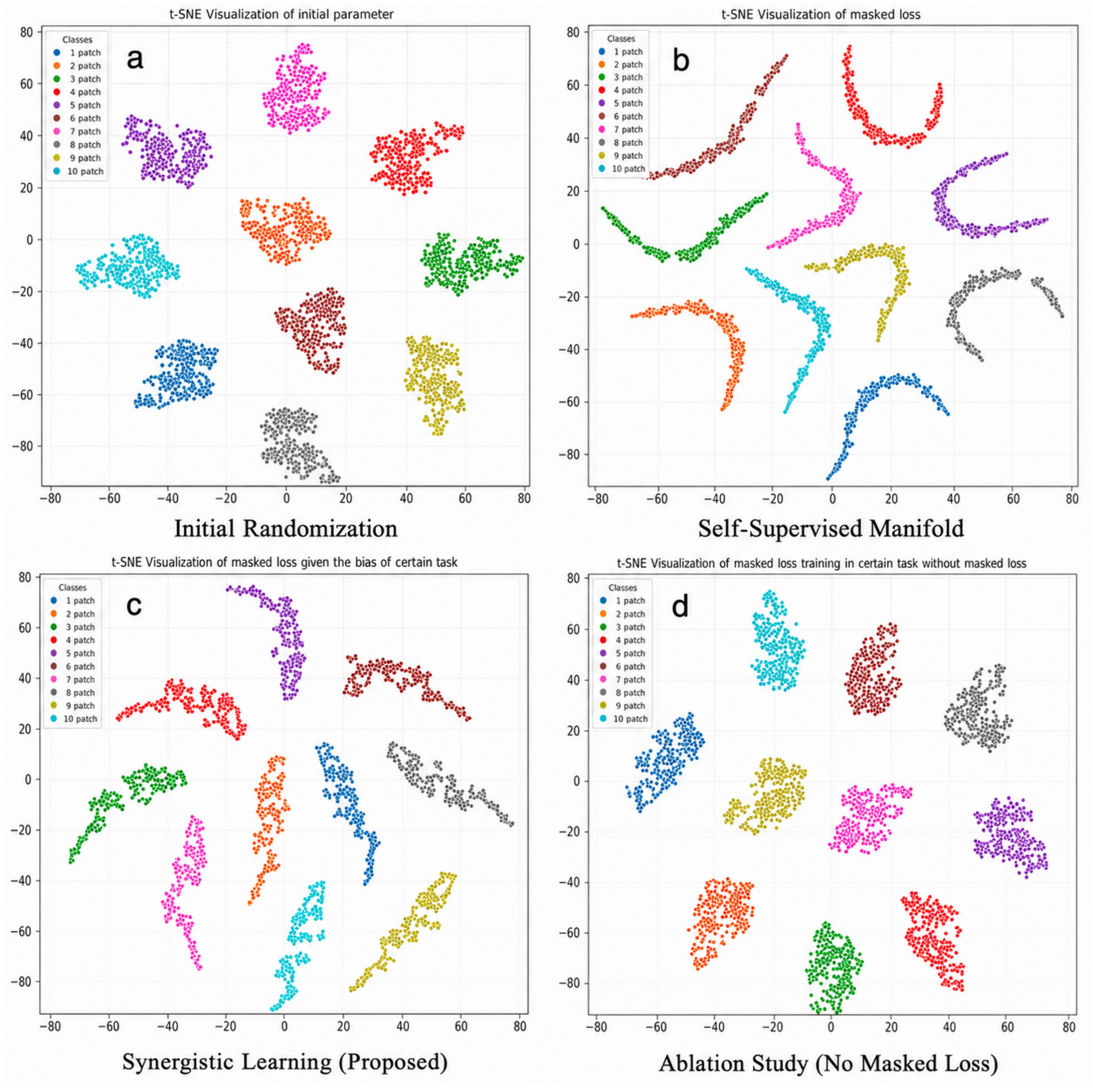
Masked consistency reconstructs low-dimensional topology in latent space (t-SNE visualization). t-SNE plots of learned latent representations for the TCGA-LGG cohort under different training regimes: (a) under initial random parameters, the feature space exhibits an isotropic, high-entropy distribution (unstructured cloud), lacking any discernible biological topology. (b) Without task interference, the Masked Loss drives disordered patches to condense into distinct curvilinear trajectories. This confirms the model successfully reconstructs underlying low-dimensional manifolds, capturing dominant non-linear variables (e.g. biological gradients) that govern data patterns. (c) Incorporating downstream task supervision ‘unfolds’ the compact manifolds created by the Masked Loss. This expands inter-sample distances along the manifold structure to maximize class separability while preserving the intrinsic biological continuum. (d) Training without the Masked Loss constraint fails to structure the latent space. The distribution remains diffuse and indistinguishable from the random initialization in (a), confirming that supervised labels alone are insufficient to reconstruct the biological topology.

To mechanistically interpret OmicsTransformer’s generalization behaviour, we visualized internal representations using gradient-based feature attribution (Fig. 6) and t-SNE dimensionality reduction (Fig. 7) on the LGG mRNA dataset.

*Masked consistency induces sparse, structured feature utilization*. Embedding projectors are initialized with near-uniform random weights (Fig. 6a). After training with the masked consistency loss, the weight distribution becomes markedly sparser with prominent peaks at a small subset of features (Fig. 6b), indicating that the model concentrates representational capacity on the most informative molecular signals. Removing the masked loss (NO MASK) prevents this sparsification and produces diffuse, noise-like weight patterns (Fig. 6c) that resemble random initialization, consistent with the observed performance degradation.

*Masked consistency unfolds biologically structured manifolds.* The geometric evolution of latent embeddings reveals the mechanism of manifold learning. Under random initialization, embeddings form a disordered cloud (Fig. 7a). Training on the supervised task alone—without masked consistency—fails to substantially reorganize this structure; data points remain diffuse and amorphous (Fig. 7d), indicating that supervised labels alone are insufficient to guide the model toward the complex internal correlation structure of omics data. Applying masked consistency without task supervision reorganizes embeddings into distinct, slender curvilinear trajectories (Fig. 7b), confirming that the objective identifies latent biological gradients—continuous variables such as tumour progression state—that govern data structure non-linearly and filter out isotropic noise. Combining masked consistency with task supervision (Fig. 7c) unfolds these compact manifolds: the curvilinear structure is preserved (maintaining biological fidelity) while data separate along discriminative boundaries (enabling precise classification). This two-stage unfolding—first denoise, then discriminate—provides geometric evidence for the mechanistic role of the masked consistency objective in OmicsTransformer’s performance.

### 3.8 Interpretability and biomarker discovery

We translated predictive manifold structure into candidate biomarkers using variance-weighted activation attribution and ensemble stability selection. By distinguishing robust gradient drivers—identified through union aggregation across ensemble runs—from a minimum essential core identified through intersection aggregation, we prioritized high-confidence biomarkers for both diagnostic and prognostic tasks (Tables 5–8, available as supplementary data at *Bioinformatics* online).

For BRCA subtyping, core markers included PRDM13, CXorf61, and a FOXA1-associated DNA methylation probe, while gradient drivers extended this set to include LIN28B and GABRA5. For LGG grading, histone cluster genes (HIST1H1B, HIST1H3B, HIST1H3C) were prominent gradient drivers, with HOXD12 and FOXA2 among the most reproducible core markers. For ROSMAP, gradient drivers included SRSF3, while core markers prioritized the epigenetic probe cg12981137 and the miRNA hsa-miR-133b.

For BRCA recurrence prediction, SYCP3 and HPSE2 emerged as stable mRNA biomarkers appearing across both gradient and core sets; somatic alterations in CDS2 and AMY2B were prioritized in the SNV modality. For BLCA, SNRPB and FBN1 ranked among the most stable SNV signals, with COL10A1 prioritized in the mRNA layer. For TARGET-WT, transcriptomic biomarkers SFRP2 and ENPP7 dominated both attribution sets. For LIHC and PRAD, the most stable SNV drivers included DNM3 and KCNQ4 (LIHC) and SRGAP3 (PRAD). Collectively, the convergence of gradient-driver and essential-core nominations across independent runs provides an internal validation that the identified biomarkers reflect reproducible predictive structure rather than single-run artefacts.

## 4 Discussion

OmicsTransformer was designed to address a central bottleneck in multi-omics learning: extracting clinically actionable signals from extremely high-dimensional, redundant, and partially missing molecular measurements. Across eight cohorts spanning diagnostic subtyping and grading, and prognostic recurrence prediction, OmicsTransformer consistently improved discrimination and run-to-run stability relative to prior multi-omics integration baselines, with the largest margins observed in prognostic tasks where signals are weak and heterogeneous. Compared with graph-based methods relying on heuristic patient similarity graphs (Wang *et al.* 2021a, Ouyang *et al.* 2023, Zhao *et al.* 2024) or architectures constrained by curated biological networks (Lan *et al.* 2024a), OmicsTransformer learns representations and cross-feature relations *de novo* through masked manifold regularization and uncertainty-aware fusion—a more flexible inductive bias for heterogeneous cancers (Melo *et al.* 2013, Chakraborty *et al.* 2018).

### 4.1 Data-driven topology versus heuristic graphs

A common assumption in multi-omics graph pipelines is that a reasonably informative patient graph can be obtained by applying cosine similarity directly to normalized, feature-selected omics vectors. In practice, omics profiles are both high-dimensional and strongly correlated (Table 1, available as supplementary data at *Bioinformatics* online), and it is well established that distance and similarity metrics concentrate in high-dimensional spaces, rendering nearest-neighbour structures unstable and highly sensitive to preprocessing choices (Aggarwal *et al.* 2001). When this occurs, constructed graphs become dense and topologically noisy, and subsequent message passing not only fails to correct these artefacts but can actively amplify them, ultimately limiting the effective resolution for clinically meaningful differences (Wang *et al.* 2021a, Ouyang *et al.* 2023, Zhao *et al.* 2024). Our observation of near-uniform hyper-similarity across raw vectors is consistent with this phenomenon and motivates learning topology only after denoising and representation learning, rather than imposing it a priori.

OmicsTransformer replaces heuristic graph construction with data-driven topology learned entirely in latent space. The relation-aware embedding module treats each modality as an unordered feature set and maps raw inputs into multiple latent tokens via learned projectors, in a manner compatible with permutation-invariant set attention (Lee *et al.* 2019). The Exponential Cosine Consistency Loss then enforces that masked and unmasked inputs yield semantically consistent embeddings—an objective closely related to denoising autoencoders and masked autoencoding that suppress feature-level noise by recovering stable low-dimensional structure from corrupted inputs (Vincent *et al.* 2008, He *et al.* 2022). The downstream Transformer subsequently models higher-order dependencies globally through self-attention (Vaswani *et al.* 2017), allowing the topology of the latent space—which features co-vary, which samples cluster—to emerge from the representation itself rather than from an external similarity heuristic. A practical consequence is linear scaling in both *N* and *D*, in contrast to the *O*(*N*^2^·*D*) pairwise-similarity cost of graph-construction pipelines.

### 4.2 Superiority in prognosis: breaking the ‘Closed-World’ limit

Prognosis differs fundamentally from diagnosis because the prediction target reflects long-term evolutionary and treatment-mediated outcomes rather than a contemporaneous molecular state. Recurrence is shaped by clonal evolution, microenvironmental selection, and therapy-induced bottlenecks, so predictive signals can be diffuse, context-dependent, and partially latent (Greaves and Maley 2012). This explains why many models that perform well on subtyping deliver smaller gains on recurrence. In this setting, OmicsTransformer’s improvements—particularly on TCGA-LIHC and TCGA-BLCA—suggest that representation-level denoising and confidence-aware fusion are essential to prevent noisy modalities or spurious correlations from dominating the integrated prediction (Table 5).

Pathway-informed architectures such as DeepKEGG encode biological prior knowledge by wiring the model to curated pathway graphs. While this improves interpretability, it also imposes a closed-world constraint: only interactions present in the knowledge base can be expressed (Lan *et al.* 2024a). Curated pathway resources are systematically incomplete and biased toward well-characterized genes; pathway analysis itself faces long-standing challenges related to context specificity, gene-set redundancy, and missing biology (Kanehisa and Goto 2000, Khatri *et al.* 2012). Critically, recurrence-relevant determinants often involve non-coding regulation, epigenetic state transitions, or ectopic developmental programs that are incompletely represented in fixed pathway diagrams (Jones and Baylin 2007, Anastasiadou *et al.* 2018). By operating in an open-world mode—with no pre-imposed pathway wiring—and learning correlations directly from cohort data, OmicsTransformer can exploit such non-canonical signals. This is consistent with both the observed performance margins over DeepKEGG and the biologically interpretable, non-canonical biomarker profiles recovered from Table 8, available as supplementary data at *Bioinformatics* online.

### 4.3 Mechanistic interpretation: manifold transformation and biological gradients

The curvilinear, low-dimensional trajectories observed in Fig. 7 support a denoising-manifold interpretation of masked consistency training. Denoising objectives are known to contract representations toward high-density regions of the data distribution, thereby learning the underlying manifold geometry and filtering isotropic noise (Vincent *et al.* 2008, Bengio *et al.* 2013). In OmicsTransformer, cosine consistency aligns masked and unmasked embeddings, encouraging each latent token to be reconstructable from correlated molecular context—i.e. from functional redundancy—rather than from feature-specific artefacts. This provides a mechanistic account of why masked training improves generalization: the model is regularized to preserve stable relational structure under realistic feature corruption.

The auxiliary patch-level classification loss further unfolds the learned manifold so that it aligns with clinically meaningful decision boundaries, preserving local continuity while enhancing global discriminability. This structure naturally supports interpretation in terms of continuous biological gradients—e.g. dedifferentiation state, immune activation level, or hypoxia score—that modulate multi-omic profiles non-linearly across patients (Jones and Baylin 2007, Melo *et al.* 2013). The t-SNE visualizations (van der Maaten and Hinton 2008) are qualitative, but the consistent emergence of organized low-dimensional structure, together with the ablation results from the NO MASK condition, provides convergent evidence that the self-supervised constraint—rather than supervised labels alone—is the primary driver of this geometric organization.

### 4.4 Robustness to missing data in clinical scenarios

From a translational standpoint, incomplete assay coverage is the rule rather than the exception: tissue availability, sequencing cost, and platform heterogeneity all contribute to systematic missingness that can correlate with clinical covariates. OmicsTransformer addresses this through uncertainty-weighted fusion, which learns a sample-specific confidence score for each modality and weights contributions accordingly. This approach is conceptually aligned with uncertainty-aware deep learning, where predictive confidence and epistemic uncertainty are used to modulate downstream decisions and reduce over-reliance on unreliable inputs (Gal and Ghahramani 2016, Kendall and Gal 2017). During training, modality dropout exposes the model to realistic missing-modality patterns, in the spirit of multimodal dropout strategies such as ModDrop that build robustness to modality absence at inference time (Neverova *et al.* 2016). The performance gains under simulated missing-modality conditions (Table 7) indicate that OmicsTransformer learns redundant cross-modal cues rather than collapsing onto a single dominant assay—a critical property for clinical deployability across heterogeneous cohorts.

### 4.5 Interpretability: from black box to biological insight

Interpretability in multi-omics is intrinsically challenging because raw feature spaces are simultaneously high-dimensional, strongly correlated, and confounded by technical effects and cell-type composition. As a consequence, naive weight inspection or single-run saliency maps can yield unstable feature rankings that are difficult to reproduce or translate into actionable hypotheses. Furthermore, attention weights are not guaranteed to constitute faithful explanations of model decisions, motivating attribution schemes that directly quantify input-feature contributions to the output (Sundararajan *et al.* 2017, Jain and Wallace 2019).

We therefore pair variance-weighted activation attribution with ensemble stability selection (Meinshausen and Bühlmann 2010). By weighting attributions according to their across-sample variance, the method down-weights features operating in saturated regimes—such as those in the flat region of the ELU activation—and highlights features that consistently separate regions of the learned manifold (Clevert *et al.* 2016). Repeating training across 20 independent runs yields two complementary biomarker sets: a Minimum Essential Core (intersection across runs) that captures stable lineage and identity anchors, and Robust Gradient Drivers (union across runs) that capture continuous progression and state factors (Tables 5–8, available as supplementary data at *Bioinformatics* online).

#### 4.5.1 Diagnostic signatures: epigenetic memory, chromatin state, and infection-associated signals

TCGA-BRCA (Table 5, available as supplementary data at *Bioinformatics* online). The Essential Core is cross-modal and coherent with established breast lineage biology. FOXA1 and accompanying methylation anchors (ZNF671, TMEFF1, MIR124-2) recur alongside an mRNA core comprising PRDM13 and the cancer-testis antigen CXorf61. FOXA1 is a pioneer transcription factor central to luminal identity and endocrine response (Seachrist *et al.* 2021), while CXorf61/KK-LC-1 has been proposed as an immunotherapy target and associated with aggressive tumour behaviour (Paret *et al.* 2015, Marcinkowski *et al.* 2019). The miRNA core is enriched for C19MC members, reported to mark and potentially drive triple-negative phenotypes and therapy resistance (Jinesh *et al.* 2018); gradient drivers such as LIN28B additionally point to a continuous dedifferentiation and stemness axis traversing the subtype landscape (Qi *et al.* 2022).

TCGA-LGG (Table 6, available as supplementary data at *Bioinformatics* online). Grade prediction is dominated by developmental regulators (HOXD12, FOXA2), chromatin-state markers (HIST1H1B, HIST1H3B, HIST1H3C), and immune activation signals (CD70). HOXD12 programs have been associated with aggressive oligodendroglioma subtypes and worse overall survival (Nuechterlein *et al.* 2024), while the prominence of histone cluster genes suggests that grade separation is partly encoded in global chromatin remodelling and proliferation-associated nucleosome dynamics. The methylation core includes MIR210, a hypoxia-responsive locus, while the miRNA core contains miR-196a-1, a microRNA repeatedly implicated in glioma progression. Together, these features indicate that hypoxia signalling and differentiation state provide a stable epigenetic backbone for grading, while immune activation via CD70 contributes along the grade-progression gradient (Lai *et al.* 2014, Guan *et al.* 2015, Seyfrid *et al.* 2022).

ROSMAP (Table 7, available as supplementary data at *Bioinformatics* online). The model prioritizes RNA-processing and DNA-repair signals: SRSF3 and MGMT methylation sites (cg12981137, cg13044136) are consistent with evidence that splicing dysregulation and impaired DNA damage responses contribute to neurodegeneration (Madabhushi *et al.* 2014, Scotti and Swanson 2016, Nussbacher *et al.* 2019). The Essential Core also includes hsa-miR-133b, reported as a serum-based diagnostic biomarker with neuroprotective effects in Alzheimer’s disease models (Yang *et al.* 2019), and an HSV-1-encoded microRNA (hsv1-miR-H1). This is notable because HSV-1 infection and reactivation have been repeatedly associated with dementia risk, and the virus encodes functional microRNAs including miR-H1 (Wozniak *et al.* 2007, Barrozo *et al.* 2020), raising the possibility that viral-host regulatory interactions contribute to the Alzheimer’s disease molecular phenotype.

#### 4.5.2 Prognostic recurrence signatures: open-world drivers beyond canonical pathways

Across recurrence cohorts (Table 8, available as supplementary data at *Bioinformatics* online), Essential Cores are compact and mechanistically interpretable, and consistently include features not represented in standard pathway catalogues. In TCGA-BRCA, the meiosis-associated cancer-testis gene SYCP3 and HPSE2 form the strict recurrence core. SYCP3 can impair mitotic homologous recombination by binding BRCA2 and inducing chromosomal instability, providing a direct molecular link to recurrence risk through genome maintenance failure (Hosoya *et al.* 2012). In TCGA-BLCA, COL10A1 appears as a gradient driver consistent with extracellular-matrix remodelling and invasive progression, while SNRPB (a core spliceosomal component) and FBN1 constitute a mutation-level essential core, implicating RNA processing fidelity and stromal architecture as independent recurrence-relevant axes (Wu *et al.* 2022, Wang *et al.* 2023). In TCGA-LIHC, the stable mRNA core (FZD9, TLE3) and SNV core (DNM3, KCNQ4) nominate Wnt-related transcriptional programmes alongside membrane-trafficking and dynamin-mediated signalling; DNM3 has been reported as a tumour suppressor in hepatocellular carcinoma, and miR-107 is an established regulator of HCC proliferation and recurrence (Inokawa *et al.* 2013, Zhang *et al.* 2015). In TCGA-PRAD, EPB41L4B/EHM2 appears in the SNV core and has been independently reported as a prognostic marker for biochemical recurrence (Wang *et al.* 2006). In TARGET-WT, the core transcriptomic features ENPP7 and SFRP2, together with the gradient miRNA miR-222, converge on developmental and Wnt-antagonist circuitry, consistent with the central role of Wnt dysregulation and extracellular pathway antagonists in Wilms tumour biology (Perotti *et al.* 2013).

These results collectively illustrate three interpretability advantages of the proposed attribution framework. First, reproducibility: the core-versus-driver distinction (intersection and union aggregation over 20 independent runs) separates stable identity anchors from continuous biological state gradients in a principled, data-driven manner. Second, cross-modal coherence: recurring cores typically couple regulatory layers (DNA methylation, miRNA) with molecular effectors (mRNA, somatic SNV), a pattern consistent with underlying gene-regulatory architecture rather than incidental statistical correlations. Third, open-world discovery: the model surfaces mechanistically plausible but non-canonical hypotheses—cancer-testis antigen involvement, spliceosomal mutation, and viral microRNA signals—that would not emerge from pathway-restricted analyses, converting latent manifold structure into prioritized, experimentally testable candidates.

### 4.6 Limitations and future directions

Several important limitations should be acknowledged. First, although we benchmarked across eight cohorts, external validation across independent centres and sequencing platforms is required to quantify generalization under realistic dataset shift; for any clinical deployment scenario, uncertainty calibration under distributional change is particularly critical (Quiñonero-Candela *et al.* 2009, Guo *et al.* 2017, Ovadia *et al.* 2019). Second, we framed prognosis as binary recurrence classification. Future work should extend to time-to-event modelling that accounts for censoring and competing risks, e.g. through Cox-based survival objectives or modern deep survival architectures (Cox 1972). Third, the current inter-modality fusion relies on a relatively simple confidence-weighting mechanism; richer cross-modal interactions—such as methylation-mediated transcriptional repression or mutation-expression coupling—may benefit from explicit cross-attention or structured biological priors. Finally, attribution-based biomarker nominations reflect statistical association rather than causation; integrating perturbation data, longitudinal profiling, or matched pre- and post-treatment samples would substantially strengthen mechanistic claims and support experimental prioritization.

Scaling behaviour with data size. Because self-attention operates over K latent patches rather than raw features or patient pairs, its cost is independent of both input dimensionality D and cohort size N. Substantially larger input spaces—raw transcriptomes (∼104) or unfiltered CpG arrays (∼105–106)—therefore remain tractable, with only the patch-embedding layer scaling linearly in D. Training cost likewise scales linearly in N, in principle supporting single-cell multi-omic cohorts with ≥105 cells. In the high-D regime, the principal challenge shifts from architectural feasibility to optimization: as the uninformative-feature fraction grows, the masked consistency objective acts increasingly as implicit feature selection (Fig. 6b). Systematic empirical profiling across D and N, including a dedicated single-cell benchmark, is an important direction for future work.

### 4.7 Conclusion

OmicsTransformer demonstrates that self-supervised manifold regularization combined with uncertainty-aware fusion constitutes a practical and principled alternative to both raw-similarity graph construction and pathway-restricted modelling. By learning topology and cross-modal integration directly from data—without relying on heuristic graph thresholds or the completeness of curated annotations—the framework delivers strong and stable performance across a broad spectrum of diagnostic and prognostic tasks, with particularly large gains in recurrence prediction where existing approaches underperform. The integrated interpretability module yields reproducible, cross-modally coherent biomarker signatures that extend beyond canonical pathway boundaries, providing a concrete foundation for hypothesis generation and downstream experimental validation in precision oncology.

## Supplementary Material

btag468_Supplementary_Data

## Data Availability

Availability of data and materials Omics data for BRCA, LGG, and the grade information for LGG dataset were obtained from The Cancer Genome Atlas Program (TCGA) through Broad GDAC Firehose (https://gdac.broadinstitute.org/). PAM50 breast cancer subtypes of BRCA dataset were obtained from TCGA biolinks (https://bioconductor.org/packages/release/bioc/vignettes/TCGAbiolinks/inst/doc/subtypes.html). Cancer prognosis omic data PRAD, BRCA, BLCA, LIHC were obtained from Xena TCGA Pan-Cancer web (http://xena.ucsc.edu) with their corresponding clinical data from TCGA. The clinical data and omic data of TARGET-WT were downloaded from the GDC Data Portal (https://portal.gdc.cancer.gov/v1/). The datasets and the code used in this study are available at https://github.com/FFJXX/OmicTransformer or from Figshare at https://doi.org/10.6084/m9.figshare.31523905.
