# Supplementary material for: OmicsTransformer: self-supervised masked consistency and uncertainty-aware fusion for robust multi-omics prediction
Source: Bioinformatics. 2026 Jun 29;42(7):btag468. doi: 10.1093/bioinformatics/btag468 (PMC13384059; doi:10.1093/bioinformatics/btag468)
Supplement: btag468_Supplementary_Data [file btag468_supplementary_data.docx]

**Supplementary Information**

**Supplementary Table 1.** **Raw-feature redundancy quantified by mean pairwise cosine similarity.** For each cohort and modality, we report the mean ± standard deviation of pairwise cosine similarity across samples after preprocessing. The consistently high values indicate hyper-similarity in raw omics space, motivating topology learning in latent space rather than constructing patient graphs directly from raw similarities.

|  | Average similarity | | |
| --- | --- | --- | --- |
| Dataset | mRNA | meth | miRNA |
| BRCA | 96.09%±2.4 | 97.21%±1.9 | 96.67%±1.4 |
| LGG | 98.29%±1.1 | 99.22%±0.8 | 97.16%±1.5 |
| ROSMAP | 98.26%±1.0 | 99.51%±0.2 | 99.47%±0.2 |

**Supplementary Table 2.** **OmicsTransformer hyperparameters by cohort.** For each dataset, we list the number of attention heads, FFN expansion ratio, Transformer depth (layers), embedding dimension (d), masking rate (r) used in the consistency loss, and number of embedding patches (K).

| Dataset | Attention Head | FNN dimension expansion | Layer | Embedding dimension | Rate | Patch number |
| --- | --- | --- | --- | --- | --- | --- |
| BRCA | 16 | ×3 | 2 | 64 | 0.8 | 10 |
| LGG | 16 | ×2 | 12 | 128 | 0.5 | 10 |
| ROSMAP | 16 | ×5 | 5 | 64 | 0.5 | 5 |
| BRCA (prognosis) | 4 | ×5 | 2 | 64 | 0.5 | 10 |
| WT | 4 | ×3 | 5 | 64 | 0.9 | 5 |
| BLCA | 8 | ×2 | 2 | 64 | 0.5 | 10 |
| LIHC | 8 | ×2 | 3 | 64 | 0.5 | 10 |
| PRAD | 2 | ×2 | 2 | 64 | 0.8 | 40 |

**Supplementary Table 3. Empirical training and inference cost of OmicsTransformer on the principal cohorts.** Inference time is measured per test split at the training batch size (one fifth of each cohort’s training-set size; see Section “Implementation Details” for rationale). Runtimes reflect GPU compute only (single GPU, mixed precision); one-time data loading and preprocessing are excluded.

| Cohort | N | Input features (total across modalities) | Per-run training time | Inference time (per test split, batch = ⅕ N_train) |
| --- | --- | --- | --- | --- |
| TCGA-BRCA | 875 | 2,503 | 32.2 min | 11.07 ms |
| TCGA-LGG | 510 | 4,548 | 24.3 min | 29.45 ms |
| ROSMAP | 351 | 600 | 59 min | 16.86 ms |
| TCGA-BLCA | 408 | 2,100 | 15.7 min | 10.7 ms |
| TCGA-LIHC | 354 | 2,200 | 27.5 min | 14.5 ms |
| TCGA-PRAD | 250 | 3,600 | 21.9 min | 25.4 ms |
| TARGET-WT | 102 | 2,200 | 11.8 min | 11.3 ms |
| TCGA-BRCA (Recurrence) | 211 | 2,100 | 11.4 min | 8.5 ms |

**Supplementary Table 4.** **Tuned hyperparameters for classical machine-learning baselines.** Settings are reported for KNN (k), SVM (kernel and key parameters), Random Forest (number of trees), shallow neural network (hidden units), and logistic regression (maximum iterations), selected under the same evaluation protocol used for deep models.

| Dataset | KNN | SVM | RF | NN | LR |
| --- | --- | --- | --- | --- | --- |
| TCGA-BRCA | k=5 | Poly kernel, d=3 | n=100 | Hidden=100 | Max iter=200 |
| TCGA-LGG | k=10 | Poly kernel, d=3, C=0.1 | n=100 | Hidden=1000 | Max iter=200 |
| ROSMAP | k=50 | Poly kernel, d=5 | n=200 | Hidden=50 | Max iter=200 |
| TCGA-BRCA (Recurrence) | k=3 | Linear kernel | n=200 | Hidden=100 | Max iter=100 |
| TCGA-LIHC | k=3 | Poly kernel, d=5 | n=300 | Hidden=100 | Max iter=100 |
| TCGA-BLCA | k=50 | Linear kernel | n=200 | Hidden=100 | Max iter=100 |
| TCGA-PRAD | k=50 | Poly kernel, d=5 | n=200 | Hidden=200 | Max iter=100 |
| TARGET-WT | k=10 | Poly kernel, d=2 | n=200 | Hidden=100 | Max iter=100 |

**Supplementary Table 5. BRCA (PAM50) biomarker candidates identified by variance-weighted attribution with ensemble stability selection.** For each modality (mRNA, DNA methylation, miRNA), we report (i) Robust Gradient Drivers: patch-aligned consensus feature modules aggregated by union across 20 independent runs, and (ii) Minimum Essential Core: features retained by strict intersection across all runs. Core features represent high-confidence anchors; gradient drivers capture broader programs along the learned manifold.

| mRNA | | |
| --- | --- | --- |
| Robust Gradient Drivers | 1 | ISL2; LOC100128977; LOC100130148; SH3GL3; HORMAD1; C1QL4; MDGA2; GABRA5; PRDM13; TPSG1; CXorf61; TRIM15; CNGB1; LIN28B |
|  | 2 | ISL2; LOC100130148; SH3GL3; HORMAD1; C1QL4; MDGA2; GABRA5; PRDM13; TRIM15; TPSG1; CXorf61; PGLYRP4; CNGB1; LIN28B |
|  | 3 | ISL2; LOC100128977; SH3GL3; HORMAD1; MDGA2; GABRA5; PRDM13; TRIM15; TPSG1; CXorf61; PGLYRP4; CNGB1; MAG; LIN28B |
|  | 4 | ISL2; LOC100128977; LOC100130148; SH3GL3; C1QL4; MDGA2; GABRA5; PRDM13; TPSG1; CXorf61; TRIM15; CNGB1; MAG; LIN28B |
|  | 5 | ISL2; LOC100128977; LOC100130148; SH3GL3; HORMAD1; C1QL4; MDGA2; GABRA5; PRDM13; TPSG1; CXorf61; TRIM15; CNGB1; MAG |
|  | 6 | ISL2; LOC100128977; SH3GL3; C1QL4; PGLYRP4; MDGA2; GABRA5; PRDM13; TPSG1; CXorf61; TRIM15; CNGB1; MAG; LIN28B |
|  | 7 | LOC100128977; LOC100130148; SH3GL3; MDGA2; GABRA5; PRDM13; CXorf49B; TPSG1; CXorf61; PGLYRP4; TRIM15; CNGB1; MAG; LIN28B |
|  | 8 | ISL2; LOC100128977; LOC100130148; SH3GL3; MDGA2; GABRA5; PRDM13; CXorf49B; TPSG1; CXorf61; TRIM15; CNGB1; MAG; LIN28B |
| Minimum Essential Core | 1 | PRDM13; CXorf61 |
| DNA Methylation | | |
| Robust Gradient Drivers | 1 | MIR124-2; TNFSF13B; ZNF671; MT1DP; FOXA1; GPR37L1; FOXD1; MIR365-1; C5orf39; TMEFF1; KLHDC9; PSAT1 |
|  | 2 | MIR124-2; CLIC6; ZNF671; MT1DP; FOXA1; GPR37L1; FOXD1; MIR365-1; C5orf39; TMEFF1; KLHDC9; PSAT1 |
|  | 3 | MIR124-2; ZNF671; MT1DP; COQ3; FOXA1; GPR37L1; FOXD1; MIR365-1; C5orf39; TMEFF1; KLHDC9; PSAT1 |
|  | 4 | MIR124-2; TNFSF13B; ZNF671; MT1DP; COQ3; FOXA1; FOXD1; MIR365-1; C5orf39; TMEFF1; KLHDC9; PSAT1 |
|  | 5 | MIR124-2; TNFSF13B; ZNF671; MT1DP; COQ3; FOXA1; GPR37L1; FOXD1; MIR365-1; C5orf39; TMEFF1; PSAT1 |
| Minimum Essential Core | 1 | MIR124-2; ZNF671; TMEFF1; MT1DP; FOXA1 |
| miRNA | | |
| Robust Gradient Drivers | 1 | hsa-mir-551a; hsa-mir-190b; hsa-mir-516a-2; hsa-mir-516a-1; hsa-mir-9-3; hsa-mir-519a-1; hsa-mir-519a-2; hsa-mir-301b; hsa-mir-577; hsa-mir-522; hsa-mir-934; hsa-mir-137; hsa-mir-138-1; hsa-mir-138-2; hsa-mir-877 |
| Minimum Essential Core | 1 | hsa-mir-516a-1; hsa-mir-516a-2; hsa-mir-519a-1; hsa-mir-577; hsa-mir-522 |

**Supplementary Table 6. LGG grading biomarker candidates identified by variance-weighted attribution with ensemble stability selection.** Reported as Robust Gradient Drivers (union across 20 runs after patch alignment) and Minimum Essential Core (intersection across all runs) for mRNA, DNA methylation, and miRNA modalities.

| mRNA | | |
| --- | --- | --- |
| Robust Gradient Drivers | 1 | PTTG3P; NPR3; HIST1H1B; FOXA2; CD70; HIST1H2BO; HIST1H3B; HIST1H3C; LBX1; REN; LOC442308; IRGM; VGLL2; HOXD12 |
|  | 2 | PTTG3P; NPR3; HIST1H1B; FOXA2; CD70; HIST1H2BO; HIST1H3B; HIST1H3C; REN; LOC442308; IRGM; VGLL2; HOXD12 |
|  | 3 | PTTG3P; NPR3; HIST1H1B; FOXA2; CD70; HIST1H2BO; HIST1H3B; HIST1H3C; LBX1; REN; LOC442308; TFAP2B; IRGM; HOXD12 |
|  | 4 | PTTG3P; NPR3; HIST1H1B; FOXA2; LBX1; HIST1H2BO; HIST1H3B; HIST1H3C; REN; TFAP2B; LOC442308; IRGM; HOXD12 |
|  | 5 | PTTG3P; NPR3; FOXA2; CD70; HIST1H2BO; HIST1H3B; HIST1H3C; LBX1; REN; LOC442308; IRGM; VGLL2; HOXD12 |
|  | 6 | PTTG3P; NPR3; HIST1H1B; FOXA2; CD70; HIST1H2BO; HIST1H3B; HIST1H3C; LBX1; LOC442308; IRGM; VGLL2; HOXD12 |
|  | 7 | NPR3; HIST1H1B; FOXA2; CD70; HIST1H2BO; LBX1; HIST1H3C; TFAP2B; LOC442308; IRGM; HOXD12 |
|  | 8 | PTTG3P; NPR3; HIST1H1B; FOXA2; CD70; LBX1; HIST1H3B; HIST1H3C; REN; LOC442308; HOXD12 |
| Minimum Essential Core | 1 | HIST1H3C; HOXD12; IRGM |
|  | 2 | HOXD12; LBX1 NPR3 |
|  | 3 | HIST1H1B; HOXD12; LOC442308 |
|  | 4 | HIST1H3B; HOXD12; PTTG3P |
|  | 6 | FOXA2; HOXD12 |
| DNA Methylation | | |
| Robust Gradient Drivers | 1 | ZNF702P; MIR210 |
| Minimum Essential Core | 1 | ZNF702P; MIR210 |
| miRNA | | |
| Robust Gradient Drivers | 1 | hsa-mir-196a-1; hsa-mir-1234 |
|  | 2 | hsa-mir-3194; hsa-mir-1234 |
| Minimum Essential Core | 1 | hsa-mir-196a-1; hsa-mir-1234 |

**Supplementary Table 7. ROSMAP Alzheimer’s disease biomarker candidates identified by variance-weighted attribution with ensemble stability selection.** Reported as Robust Gradient Drivers (union across 20 runs after patch alignment) and Minimum Essential Core (intersection across all runs) for mRNA, DNA methylation, and miRNA modalities.

| mRNA | | |
| --- | --- | --- |
| Robust Gradient Drivers | 1 | MIR1302-10;RPL21;LINC02593;LINC01128;SRSF3 |
|  | 2 | MIR1302-10;LINC01871;LINC02593;LINC01128;SRSF3 |
| Minimum Essential Core | 1 | MIR1302-10;LINC02593 |
| DNA Methylation | | |
| Robust Gradient Drivers | 1 | cg07992625;cg12799265;cg12981137 |
|  | 2 | cg18001427;cg07992625;cg12799265 |
| Minimum Essential Core | 1 | cg13044136;cg12981137 |
| miRNA | | |
| Robust Gradient Drivers | 1 | hsa-miR-133b; hsa-miR-200a |
| Minimum Essential Core | 1 | hsv1-miR-H1; hsa-miR-133b |

**Supplementary Table 8. Recurrence prediction biomarker candidates across cohorts identified by variance-weighted attribution with ensemble stability selection.** For each cohort and available modality (mRNA, SNV, miRNA), we report Robust Gradient Drivers (union across 20 runs after patch alignment) and Minimum Essential Core (intersection across all runs).

**(a) BRCA**

| mRNA | | |
| --- | --- | --- |
| Robust Gradient Drivers | 1 | SYCP3;HPSE2 |
| Minimum Essential Core | 1 | SYCP3;HPSE2 |
| SNV | | |
| Robust Gradient Drivers | 1 | CDS2;AMY2B |
| Minimum Essential Core | 1 | CDS2;AMY2B |
| miRNA | | |
| Robust Gradient Drivers | 1 | hsa-mir-579;hsa-mir-570 |
| Minimum Essential Core | 1 | hsa-mir-381;hsa-mir-421 |

| mRNA | | |
| --- | --- | --- |
| Robust Gradient Drivers | 1 | COL10A1;HAP1 |
| Minimum Essential Core | 1 | HTR2B;TAS2R38 |
| SNV | | |
| Robust Gradient Drivers | 1 | SNRPB;FBN1 |
| Minimum Essential Core | 1 | SNRPB;FBN1 |
| miRNA | | |
| Robust Gradient Drivers | 1 | hsa-mir-380;hsa-mir-369 |
| Minimum Essential Core | 1 | hsa-mir-299;hsa-mir-369 |

**(b) BLCA**

**(c) LIHC**

| mRNA | | |
| --- | --- | --- |
| Robust Gradient Drivers | 1 | FZD9;TLE3 |
| Minimum Essential Core | 1 | FZD9;TLE3 |
| SNV | | |
| Robust Gradient Drivers | 1 | KCNQ4;DNM3 |
| Minimum Essential Core | 1 | KCNQ4;DNM3 |
| miRNA | | |
| Robust Gradient Drivers | 1 | hsa-mir-190a', 'hsa-mir-589 |
| Minimum Essential Core | 1 | hsa-mir-107', 'hsa-mir-199b |

**(d) PRAD**

| mRNA | | |
| --- | --- | --- |
| Robust Gradient Drivers | 1 | KLC1;SEMA5B |
| Minimum Essential Core | 1 | KLC1;SEMA5B |
| SNV | | |
| Robust Gradient Drivers | 1 | SRGAP3;EPB41L4B |
| Minimum Essential Core | 1 | SRGAP3;EPB41L4B |
| miRNA | | |
| Robust Gradient Drivers | 1 | hsa-mir-675;hsa-mir-4787 |
|  | 2 | hsa-mir-206;hsa-mir-493 |
|  | 3 | hsa-mir-493;hsa-mir-184 |
|  | 4 | hsa-mir-1258;hsa-mir-187 |
|  | 5 | hsa-mir-1291;hsa-mir-187 |
| Minimum Essential Core | 1 | hsa-mir-4286;hsa-mir-5586 |
|  | 2 | hsa-mir-3940;hsa-mir-891a |
|  | 3 | hsa-mir-137;hsa-mir-4326 |
|  | 4 | hsa-mir-3622a;hsa-mir-15b |
|  | 5 | hsa-mir-509-3;hsa-mir-493 |
|  | 6 | hsa-mir-493;hsa-mir-184 |
|  | 7 | hsa-mir-653;hsa-mir-192 |
|  | 8 | hsa-mir-449a;hsa-mir-340 |
|  | 9 | hsa-mir-885;hsa-mir-615 |
|  | 10 | hsa-mir-192;hsa-mir-1248 |

**(e) WT**

| mRNA | | |
| --- | --- | --- |
| Robust Gradient Drivers | 1 | ENPP7;SFRP2 |
| Minimum Essential Core | 1 | ENPP7;SFRP2 |
| miRNA | | |
| Robust Gradient Drivers | 1 | hsa-mir-222;hsa-mir-573 |
| Minimum Essential Core | 1 | hsa-mir-3691;hsa-mir-655 |


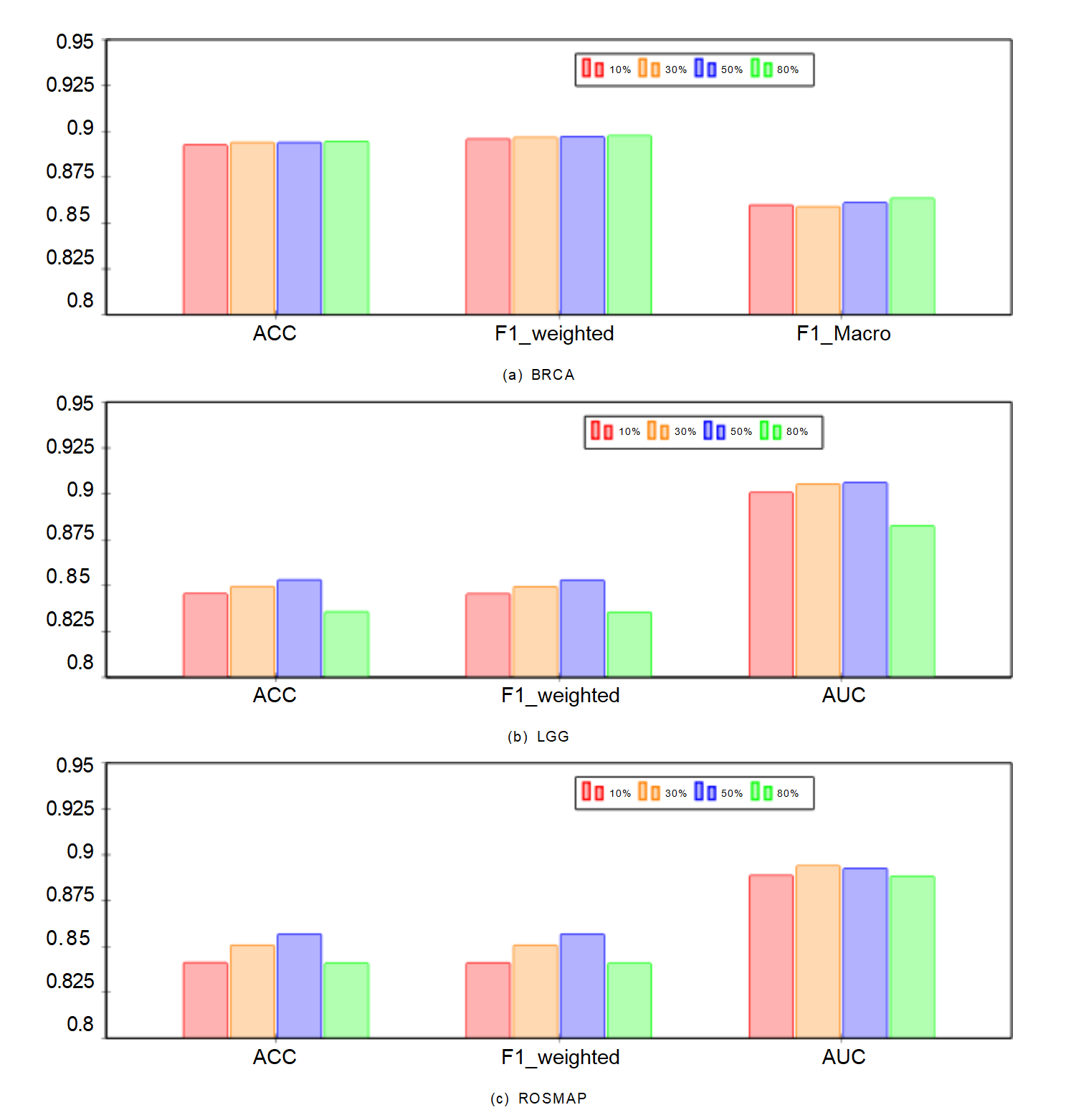


**Supplementary Figure 1. Sensitivity analysis of the masking rate (*r*).** Performance of OmicsTransformer as a function of r on (a) BRCA, (b) LGG, and (c) ROSMAP. Curves summarize mean ± standard deviation across 20 independent runs; other hyperparameters are fixed per Supplementary Table 2.


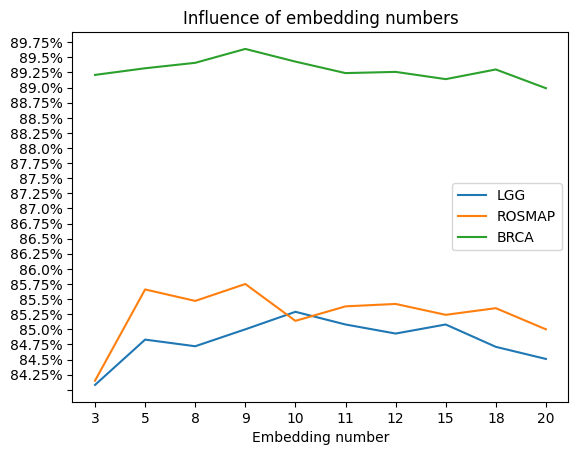


**Supplementary Figure 2. Sensitivity analysis of embedding subspace count (*K*).** Accuracy as a function of K on BRCA, LGG, and ROSMAP. Curves summarize mean ± standard deviation across 20 independent runs; other hyperparameters are fixed per Supplementary Table 2.
